# Evaluation of the metrological reliability of a graduated cylinder from experimental data from an *in-situ* calibration

**DOI:** 10.1016/j.dib.2020.106133

**Published:** 2020-08-06

**Authors:** José Daniel Hernández-Vásquez, Cristian Antonio Pedraza-Yepes, Andrés David Rodriguez-Salas, Jorge Luis Bolívar-Solana, Darío Andres Gonzalez-Camacho

**Affiliations:** a Universidad Antonio Nariño, Faculty of Mechanical, Electronic and Biomedical Engineering (FIMEB), Research Group GIFOURIER. Mechanical Engineering Program, Puerto Colombia, Colombia; b Universidad del Atlántico, Faculty of Engineering, Mechanical Engineering Program, Research Group CONFORMAT, Puerto Colombia, Colombia; c Universidad del Atlántico, Faculty of Basic Sciences, Physics Program, Research Group GIM, Puerto Colombia, Colombia

**Keywords:** Metrology, Graduated cylinder, Calibration, Volume measurement, Mass measurement

## Abstract

This work presents the data experimentally collected in a chemical laboratory for the calibration of a graduated cylinder. There are several factors that can influence the volume measurement using this type of instrument and, consequently, its metrological reliability, for example: the internal geometry, the environmental conditions (ambient temperature, atmospheric pressure and relative humidity), the acceleration of gravity, the density of the air, among others. For the data collection it was necessary to use a glass liquid thermometer (Range: 0–10 °C), a digital thermohygrometer (Range: 0–100 °C and 0–99%RH) and a digital barometer (Range: 0–9999 mbar). Additionally, an analytical scale (Range: 0–220 g) was used for mass measurement. From the measurements obtained, it was possible to determine the *in-situ* air density and the buoyancy factor that influences the mass measurement. The data, rigorously obtained, present a potential use to determine the metrological reliability of a graduated cylinder for laboratory use and, additionally, contribute to perform a metrological validation of alternative methods for the calibration of graduated cylinder.

**Specifications Table**

**Table:** 

Subject	Mechanical Engineering.
Specific subject area	Measurement of thermodynamic magnitudes (temperature, pressure, relative humidity, volume, mass) applied in Mechanical Engineering.
Type of data	Table.
How data were acquired	Experimental measurements performed in a chemical laboratory under specified environmental conditions. Properly calibrated and certified measuring instruments were used: analytical scale, graduated cylinder, glass liquid thermometer, thermohygrometer and barometer.
Data format	Raw and Analyzed.
Parameters for data collection	The ambient temperature must not exceed 25 °C. The analytical balance must be located on a flat surface, balanced and free from drafts, as well as from vibrations. In addition, it must wait 25 min for its use. At this point an electronic balance of its parts is expected. Distilled water should be used at room temperature. Additionally, the standard masses must be thermally equilibrated with the ambient temperature. For this they should be used after 30 min.
Description of data collection	Once the first calibration of the digital scale was completed, the graduated cylinder was calibrated. Pure water was used for this activity and the total volume of the graduated cylinder was achieved by introducing small volumes (approximately 0.5 ml in 0.5 ml). At each experimental point the water temperature was measured with the help of the liquid glass thermometer. Additionally, atmospheric pressure and ambient temperature were measured (manufacturer: Forecast; Range: 0 a 9999 mbar; Resolution: 1 mbar). The objective of increasing the volume of the graduated cylinder in small volumes was to reduce the impact of non-homogeneity related to the internal diameter. Once the calibration of the balance was finished, the calibration of the graduated cylinder was carried out without discounting its initial mass. The graduated cylinder was calibrated, 91 experimental points were obtained, to total the maximum volumetric capacity of 50 ml.
Data source location	Universidad del Atlántico, Barranquilla, Colombia.
Data accessibility	With the article

Value of the Data

• Documented data is particularly important because it allows an in-depth assessment of the contribution of error and uncertainty of the tare function to the mass measurement process.
• The published data is very important for professionals in the physical, chemical and engineering areas. With this data and the applied methodology, they can assess the metrological reliability of not only scales, but also electronic devices for measuring temperature, pressure and other digital instruments.
• From the published data it is possible to apply other statistical methods associated with the uncertainty analysis (example: Monte Carlo method) and to evaluate the potentiality of these methods applied to electronic devices used in a laboratory.
• In the medium term, the results of similar research, using published data, are expected to contribute to improving the reliability of measurements for drug manufacturing. Furthermore, in this era where high metrological reliability of measurement equipment is required to investigate a vaccine against covid-19.
• As an added value, the published data was obtained by applying high scientific rigor, using measurement standards traceable to the International System of Units. This becomes an unquestionable reliability of the results and discussions that can be obtained by applying analytical methods.

## 1 Data description

This section presents the experimental data, as: (i) evaluate the influence of the tare function on the metrological reliability of a scale and (ii) know the impact that the tare function introduces on the uncertainty and errors associated with the volume measurement, using a graduated cylinder.

### 1.1 Calibration of the analytical scale applying the tare function

According to the methodology described in the before section, Table 1 presents the experimental data obtained when the Tare Function was applied in the calibration analytical scale process. This table shows 204 experimental points (102 in ascending load and 102 in descending load) and the standard mass value which was increasing each 0.5 g. The mass indicated by the scale was a negative value. It is due to the original mass of the graduated cylinder (73.6239 g) was discounted. The environmental temperature and the atmospheric pressure were measured for each experimental point. Due to the number of experimental points obtained, it is possible to carry out a deeper analysis around the operating range of the balance. In other words, the data presented in Table 1 allow intermediate measurement points with low measurement uncertainty to be found. The literature has various methods that can be used for the statistical treatment of data, for example, the method of ordinary least squares.

**Table 1:** Experimental data (applying the Tare Function).

Exp. Points	Standard Mass	ASCENDING LOAD			DESCENDING LOAD		
		Mass indicated by the scale	Environmental Temperature	Atmospheric Pressure	Mass indicated by the scale	Environmental Temperature	Atmospheric Pressure
	g	g	°C	mbar/abs	g	°C	mbar/abs
1	0.0	−73.6239	32.8	1001	−73.6145	29.8	1000
2	0.5	−73.1240	32.8	1001	−73.1155	30.4	1002
3	2.5	−71.1238	32.5	1001	−71.1165	30.4	1002
4	4.5	−69.1240	32.0	1001	−69.1166	30.4	1002
5	6.5	−67.1233	31.7	1001	−67.1175	30.4	1002
6	8.5	−65.1234	31.5	1001	−65.1179	30.4	1002
7	10.5	−63.1233	31.4	1002	−63.1175	30.4	1002
8	12.5	−61.1235	31.3	1001	−61.1182	30.4	1002
9	14.5	−59.1237	31.3	1002	−59.1178	30.4	1002
10	16.5	−57.1233	31.2	1002	−57.1189	30.4	1002
11	18.5	−55.1236	31.2	1002	−55.1192	30.4	1002
12	20.5	−53.1231	31.2	1002	−53.1204	30.4	1001
13	22.5	−51.1234	31.1	1002	−51.1199	30.4	1001
14	24.5	−49.1232	31.1	1002	−49.1206	30.4	1001
15	26.5	−47.1232	31.1	1002	−47.1210	30.4	1001
16	28.5	−45.1233	31.1	1002	−45.1212	30.4	1001
17	30.5	−43.1233	31.0	1002	−43.1210	30.4	1001
18	32.5	−41.1235	31.0	1002	−41.1215	30.4	1001
19	34.5	−39.1233	30.9	1002	−39.1229	30.4	1001
20	36.5	−37.1235	30.9	1002	−37.1227	30.4	1001
21	38.5	−35.1232	30.9	1002	−35.1223	30.4	1001
22	40.5	−33.1217	30.9	1002	−33.1230	30.4	1001
23	42.5	−31.1218	30.8	1002	−31.1220	30.4	1001
24	44.5	−29.1215	30.8	1002	−29.1229	30.4	1001
25	46.5	−27.1216	30.8	1002	−27.1229	30.4	1001
26	48.5	−25.1216	30.8	1002	−25.1230	30.4	1001
27	50.5	−23.1216	30.7	1002	−23.1230	30.4	1001
28	52.5	−21.1217	30.7	1002	−21.1228	30.4	1001
29	54.5	−19.1219	30.7	1002	−19.1232	30.4	1001
30	56.5	−17.1218	30.6	1002	−17.1229	30.4	1001
31	58.5	−15.1218	30.6	1002	−15.1227	30.4	1001
32	60.5	−13.1218	30.5	1002	−13.1230	30.4	1001
33	62.5	−11.1219	30.5	1002	−11.1230	30.4	1001
34	64.5	−9.1216	30.5	1002	−9.1229	30.4	1001
35	66.5	−7.1220	30.5	1002	−7.1229	30.4	1001
36	68.5	−5.1220	30.5	1002	−5.1230	30.4	1001
37	70.5	−3.1223	30.5	1002	−3.1229	30.4	1001
38	72.5	−1.1220	30.5	1002	−1.1228	30.4	1001
39	74.5	0.8780	30.4	1002	0.8772	30.4	1001
40	76.5	2.8777	30.4	1002	2.8770	30.4	1001
41	78.5	4.8775	30.4	1002	4.8773	30.4	1001
42	80.5	6.8772	30.4	1002	6.8771	30.4	1001
43	82.5	8.8773	30.4	1002	8.8769	30.4	1001
44	84.5	10.8774	30.4	1002	10.8773	30.4	1001
45	86.5	12.8778	30.4	1002	12.8770	30.4	1002
46	88.5	14.8776	30.4	1002	14.8766	30.4	1002
47	90.5	16.8777	30.4	1002	16.8770	30.4	1002
48	92.5	18.8777	30.4	1002	18.8770	30.4	1002
49	94.5	20.8779	30.4	1002	20.8768	30.4	1002
50	96.5	22.8775	30.4	1002	22.8770	30.4	1002
51	98.5	24.8775	30.4	1002	24.8771	30.4	1002
52	100.5	26.8766	30.3	1002	26.8760	30.4	1002
53	102.5	28.8765	30.3	1002	28.8761	30.3	1002
54	104.5	30.8766	30.3	1002	30.8758	30.3	1002
55	106.5	32.8764	30.3	1002	32.8759	30.3	1002
56	108.5	34.8765	30.3	1002	34.8760	30.3	1002
57	110.5	36.8765	30.3	1002	36.8756	30.3	1002
58	112.5	38.8766	30.3	1002	38.8757	30.3	1002
59	114.5	40.8766	30.3	1002	40.8757	30.2	1002
60	116.5	42.8766	30.3	1002	42.8758	30.2	1002
61	118.5	44.8765	30.3	1002	44.8762	30.2	1002
62	120.5	46.8766	30.3	1002	46.8758	30.2	1002
63	122.5	48.8765	30.3	1002	48.8757	30.2	1002
64	124.5	50.8763	30.3	1002	50.8758	30.2	1002
65	126.5	52.8763	30.2	1002	52.8759	30.2	1002
66	128.5	54.8763	30.2	1002	54.8761	30.2	1002
67	130.5	56.8762	30.2	1002	56.8756	30.2	1002
68	132.5	58.8761	30.2	1002	58.8754	30.2	1002
69	134.5	60.8762	30.2	1002	60.8756	30.2	1002
70	136.5	62.8762	30.2	1002	62.8754	30.2	1002
71	138.5	64.8760	30.2	1002	64.8754	30.2	1002
72	140.5	66.8758	30.2	1002	66.8758	30.2	1002
73	142.5	68.8762	30.2	1002	68.8758	30.2	1002
74	144.5	70.8762	30.2	1002	70.8756	30.2	1002
75	146.5	72.8760	30.2	1002	72.8755	30.2	1002
76	148.5	74.8759	30.2	1002	74.8754	30.2	1002
77	150.5	76.8761	30.2	1002	76.8755	30.2	1002
78	152.5	78.8759	30.2	1002	78.8757	30.2	1002
79	154.5	80.8761	30.2	1002	80.8757	30.2	1002
80	156.5	82.8760	30.2	1002	82.8757	30.2	1002
81	158.5	84.8760	30.2	1002	84.8752	30.2	1002
82	160.5	86.8758	30.2	1002	86.8758	30.2	1002
83	162.5	88.8759	30.2	1002	88.8755	30.2	1002
84	164.5	90.8758	30.2	1002	90.8755	30.2	1002
85	166.5	92.8757	30.2	1002	92.8755	30.2	1002
86	168.5	94.8757	30.2	1002	94.8753	30.2	1002
87	170.5	96.8760	30.2	1002	96.8753	30.2	1002
88	172.5	98.8762	30.2	1002	98.8755	30.2	1002
89	174.5	100.8760	30.2	1002	100.8754	30.2	1002
90	176.5	102.8760	30.2	1002	102.8753	30.2	1002
91	178.5	104.8759	30.2	1002	104.8754	30.2	1002
92	180.5	106.8757	30.1	1002	106.8753	30.2	1002
93	182.5	108.8760	30.1	1002	108.8753	30.2	1002
94	184.5	110.8761	30.1	1002	110.8750	30.2	1002
95	186.5	112.8760	30.1	1002	112.8752	30.2	1002
96	188.5	114.8761	30.1	1002	114.8753	30.2	1002
97	190.5	116.8759	30.1	1002	116.8751	30.2	1002
98	192.5	118.8758	30.1	1002	118.8755	30.2	1002
99	194.5	120.8759	30.1	1002	120.8755	30.2	1002
100	196.5	122.8757	30.1	1002	122.8755	30.2	1002
101	198.5	124.8759	30.1	1002	124.8758	30.2	1002
102	200.5	126.8753	30.1	1002	126.8753	30.1	1002

Table 2 presents the experimental data. Applying the uncertainty expansion theory [1], [2], [3], [4] and using the certificate of calibration [5] was possible to calculate the uncertainty associated to the standard mass. Moreover, Table 2 shows, in highlight, the indicated mass by the analytical scale, the apparent mass and its uncertainty. In relation to the measurement uncertainty, it can be seen that the values associated with the standard mass are in the order of micrograms. This confirms that the metrological hierarchy of the mass standards used to obtain the data. In relation to the measurement uncertainty associated with temperature, it can be seen that the obtained value of 0.029 °C is at least three times less than the resolution of the instrument used. This confirms the high accuracy associated with the temperature measurement process. Finally, the calculated value for air density (1.14 kg/m^3^) is in line with the average values found in the literature (approximately 1.2 kg/m^3^) and its measurement uncertainty is much less than the tabulated values. and published in the literature, approximately equal to 5% of the measured value (i.e.: 0.06 kg/m^3^). Table 2 shows that the uncertainty obtained for the air density varies between 0.00069 kg/m^3^ to 0.00070 kg/m^3^. Unquestionably, this confirms the scientific rigor with which the experiments have been carried out and, consequently, the obtaining of the data.

The environmental temperature and the atmospheric pressure were measured using calibrated instruments. The certificate of calibration reports the uncertainty in 0.029 °C for the thermometer and 0.60 mbar/abs for the barometer [5]. This data are showed in the ascending and descending load situations.

**Fig. 1:**
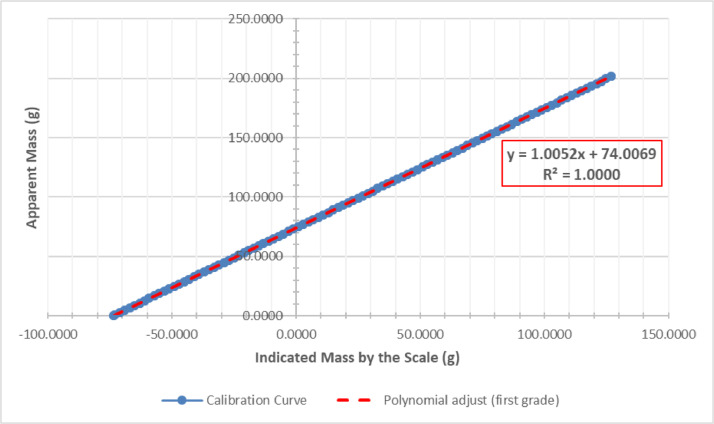
Polynomial adjustment curve (first degree polynomial).

Once the dataset in the previous table were obtained, the method of ordinary least squares was applied, whose foundations are detailed in other works [6], [7], [8], [9]. This method allowed establishing a relationship between the mass indicated by the analytical scale (horizontal axis) and the apparent mass calculated (vertical axis). In this way, the uncertainty of the adjustment (***u***_***f***_) of four different polynomials (grade 1, 2, 3 and 4) was evaluated. It was obtained that the first degree polynomial is the one that best represents the physical nature of the problem, once its uncertainty of adjustment (i.e.:, the uncertainty that adds to the calibration process the fact of making an adjustment polynomial) turned out to be the least of all. The uncertainty value of the adjustment is equal to 0.0028 g. Fig. 1 shows the calibration curve obtained, as well as the adjustment equation. In this figure we can see the coefficients of the first-degree fit equation (*y = axe + b*) where a corresponds to the slope (*a* = 1.0052) and b corresponds to the intercept with the vertical axis (*b* = 74.0069). The determination coefficient R2 represents the agreement between the mathematical model and the analyzed physical phenomenon. The obtained value (R^2^ = 1.0000) is a clear example of the excellent relationship between the mathematical model and the physical model.

The expanded uncertainty associated to mass measurement is calculated. Table 3 shows the dataset for the ascending and descending load situations: mass indicated by the analytical scale and mass adjusted by the polynomial. In addition, The uncertainty analysis, for each experimental point, is shown.

**Table 3:** Uncertainty analysis (applying the tare function).

Exp. Points	ASCENDING LOAD		DESCENDING LOAD		UNCERTAINTY ANALYSIS				
	Indicated Mass	Ajusted Mass	Indicated Mass	Ajusted Mass	Adjusted Uncertainty	Standard Mass Uncertainty	Resolution Uncertainty	Combined Uncertainty	Expanded Uncertainty
	g	G	g	g	g	g	g	g	g
1	−73.6239	0.0003	−73.6145	0.0097	0.00280	0.0000000	0.000029	0.0028	0.0055
2	−73.1240	0.5028	−73.1155	0.5113	0.00280	0.0000042	0.000029	0.0028	0.0055
3	−71.1238	2.5134	−71.1165	2.5207	0.00280	0.0000077	0.000029	0.0028	0.0055
4	−69.1240	4.5236	−69.1166	4.5310	0.00280	0.0000101	0.000029	0.0028	0.0055
5	−67.1233	6.5347	−67.1175	6.5405	0.00280	0.0000107	0.000029	0.0028	0.0055
6	−65.1234	8.5450	−65.1179	8.5505	0.00280	0.0000125	0.000029	0.0028	0.0055
7	−63.1233	10.5555	−63.1175	10.5613	0.00280	0.0000108	0.000029	0.0028	0.0055
8	−61.1235	12.5657	−61.1182	12.5710	0.00280	0.0000126	0.000029	0.0028	0.0055
9	−59.1237	14.5759	−59.1178	14.5818	0.00280	0.0000142	0.000029	0.0028	0.0055
10	−57.1233	16.5867	−57.1189	16.5911	0.00280	0.0000146	0.000029	0.0028	0.0055
11	−55.1236	18.5968	−55.1192	18.6012	0.00280	0.0000160	0.000029	0.0028	0.0055
12	−53.1231	20.6077	−53.1204	20.6104	0.00280	0.0000141	0.000029	0.0028	0.0055
13	−51.1234	22.6178	−51.1199	22.6213	0.00280	0.0000155	0.000029	0.0028	0.0055
14	−49.1232	24.6284	−49.1206	24.6310	0.00280	0.0000169	0.000029	0.0028	0.0055
15	−47.1232	26.6388	−47.1210	26.6410	0.00280	0.0000167	0.000029	0.0028	0.0055
16	−45.1233	28.6491	−45.1212	28.6512	0.00280	0.0000179	0.000029	0.0028	0.0055
17	−43.1233	30.6595	−43.1210	30.6618	0.00280	0.0000173	0.000029	0.0028	0.0055
18	−41.1235	32.6697	−41.1215	32.6717	0.00280	0.0000185	0.000029	0.0028	0.0055
19	−39.1233	34.6803	−39.1229	34.6807	0.00280	0.0000196	0.000029	0.0028	0.0055
20	−37.1235	36.6905	−37.1227	36.6913	0.00280	0.0000199	0.000029	0.0028	0.0055
21	−35.1232	38.7012	−35.1223	38.7021	0.00280	0.0000210	0.000029	0.0028	0.0055
22	−33.1217	40.7131	−33.1230	40.7118	0.00280	0.0000195	0.000029	0.0028	0.0055
23	−31.1218	42.7234	−31.1220	42.7232	0.00280	0.0000206	0.000029	0.0028	0.0055
24	−29.1215	44.7341	−29.1229	44.7327	0.00280	0.0000216	0.000029	0.0028	0.0055
25	−27.1216	46.7444	−27.1229	46.7431	0.00280	0.0000219	0.000029	0.0028	0.0055
26	−25.1216	48.7548	−25.1230	48.7534	0.00280	0.0000228	0.000029	0.0028	0.0055
27	−23.1216	50.7652	−23.1230	50.7638	0.00280	0.0000170	0.000029	0.0028	0.0055
28	−21.1217	52.7755	−21.1228	52.7744	0.00280	0.0000182	0.000029	0.0028	0.0055
29	−19.1219	54.7857	−19.1232	54.7844	0.00280	0.0000193	0.000029	0.0028	0.0055
30	−17.1218	56.7962	−17.1229	56.7951	0.00280	0.0000197	0.000029	0.0028	0.0055
31	−15.1218	58.8066	−15.1227	58.8057	0.00280	0.0000207	0.000029	0.0028	0.0055
32	−13.1218	60.8170	−13.1230	60.8158	0.00280	0.0000197	0.000029	0.0028	0.0055
33	−11.1219	62.8273	−11.1230	62.8262	0.00280	0.0000208	0.000029	0.0028	0.0055
34	−9.1216	64.8380	−9.1229	64.8367	0.00280	0.0000218	0.000029	0.0028	0.0055
35	−7.1220	66.8480	−7.1229	66.8471	0.00280	0.0000221	0.000029	0.0028	0.0055
36	−5.1220	68.8584	−5.1230	68.8574	0.00280	0.0000230	0.000029	0.0028	0.0055
37	−3.1223	70.8685	−3.1229	70.8679	0.00280	0.0000217	0.000029	0.0028	0.0055
38	−1.1220	72.8792	−1.1228	72.8784	0.00280	0.0000227	0.000029	0.0028	0.0055
39	0.8780	74.8896	0.8772	74.8888	0.00280	0.0000236	0.000029	0.0028	0.0055
40	2.8777	76.8997	2.8770	76.8990	0.00280	0.0000239	0.000029	0.0028	0.0055
41	4.8775	78.9099	4.8773	78.9097	0.00280	0.0000247	0.000029	0.0028	0.0055
42	6.8772	80.9200	6.8771	80.9199	0.00280	0.0000239	0.000029	0.0028	0.0055
43	8.8773	82.9305	8.8769	82.9301	0.00280	0.0000248	0.000029	0.0028	0.0055
44	10.8774	84.9410	10.8773	84.9409	0.00280	0.0000256	0.000029	0.0028	0.0055
45	12.8778	86.9518	12.8770	86.9510	0.00280	0.0000239	0.000029	0.0028	0.0055
46	14.8776	88.9620	14.8766	88.9610	0.00280	0.0000247	0.000029	0.0028	0.0055
47	16.8777	90.9725	16.8770	90.9718	0.00280	0.0000256	0.000029	0.0028	0.0055
48	18.8777	92.9829	18.8770	92.9822	0.00280	0.0000264	0.000029	0.0028	0.0055
49	20.8779	94.9935	20.8768	94.9924	0.00280	0.0000272	0.000029	0.0028	0.0055
50	22.8775	97.0035	22.8770	97.0030	0.00280	0.0000274	0.000029	0.0028	0.0055
51	24.8775	99.0139	24.8771	99.0135	0.00280	0.0000282	0.000029	0.0028	0.0055
52	26.8766	101.0234	26.8760	101.0228	0.00280	0.0000268	0.000029	0.0028	0.0055
53	28.8765	103.0337	28.8761	103.0333	0.00280	0.0000276	0.000029	0.0028	0.0055
54	30.8766	105.0442	30.8758	105.0434	0.00280	0.0000284	0.000029	0.0028	0.0055
55	32.8764	107.0544	32.8759	107.0539	0.00280	0.0000286	0.000029	0.0028	0.0055
56	34.8765	109.0649	34.8760	109.0644	0.00280	0.0000293	0.000029	0.0028	0.0055
57	36.8765	111.0753	36.8756	111.0744	0.00280	0.0000286	0.000029	0.0028	0.0055
58	38.8766	113.0858	38.8757	113.0849	0.00280	0.0000294	0.000029	0.0028	0.0055
59	40.8766	115.0962	40.8757	115.0953	0.00280	0.0000301	0.000029	0.0028	0.0055
60	42.8766	117.1066	42.8758	117.1058	0.00280	0.0000303	0.000029	0.0028	0.0055
61	44.8765	119.1169	44.8762	119.1166	0.00280	0.0000310	0.000029	0.0028	0.0055
62	46.8766	121.1274	46.8758	121.1266	0.00280	0.0000300	0.000029	0.0028	0.0055
63	48.8765	123.1377	48.8757	123.1369	0.00280	0.0000307	0.000029	0.0028	0.0055
64	50.8763	125.1479	50.8758	125.1474	0.00280	0.0000314	0.000029	0.0028	0.0055
65	52.8763	127.1583	52.8759	127.1579	0.00280	0.0000316	0.000029	0.0028	0.0055
66	54.8763	129.1687	54.8761	129.1685	0.00280	0.0000323	0.000029	0.0028	0.0055
67	56.8762	131.1790	56.8756	131.1784	0.00280	0.0000317	0.000029	0.0028	0.0055
68	58.8761	133.1893	58.8754	133.1886	0.00280	0.0000323	0.000029	0.0028	0.0055
69	60.8762	135.1998	60.8756	135.1992	0.00280	0.0000330	0.000029	0.0028	0.0055
70	62.8762	137.2102	62.8754	137.2094	0.00280	0.0000332	0.000029	0.0028	0.0055
71	64.8760	139.2204	64.8754	139.2198	0.00280	0.0000338	0.000029	0.0028	0.0055
72	66.8758	141.2306	66.8758	141.2306	0.00280	0.0000329	0.000029	0.0028	0.0055
73	68.8762	143.2414	68.8758	143.2410	0.00280	0.0000336	0.000029	0.0028	0.0055
74	70.8762	145.2518	70.8756	145.2512	0.00280	0.0000342	0.000029	0.0028	0.0055
75	72.8760	147.2620	72.8755	147.2615	0.00280	0.0000344	0.000029	0.0028	0.0055
76	74.8759	149.2723	74.8754	149.2718	0.00280	0.0000350	0.000029	0.0028	0.0055
77	76.8761	151.2829	76.8755	151.2823	0.00280	0.0000315	0.000029	0.0028	0.0055
78	78.8759	153.2931	78.8757	153.2929	0.00280	0.0000322	0.000029	0.0028	0.0055
79	80.8761	155.3037	80.8757	155.3033	0.00280	0.0000328	0.000029	0.0028	0.0055
80	82.8760	157.3140	82.8757	157.3137	0.00280	0.0000330	0.000029	0.0028	0.0055
81	84.8760	159.3244	84.8752	159.3236	0.00280	0.0000336	0.000029	0.0028	0.0055
82	86.8758	161.3346	86.8758	161.3346	0.00280	0.0000330	0.000029	0.0028	0.0055
83	88.8759	163.3451	88.8755	163.3447	0.00280	0.0000337	0.000029	0.0028	0.0055
84	90.8758	165.3554	90.8755	165.3551	0.00280	0.0000343	0.000029	0.0028	0.0055
85	92.8757	167.3657	92.8755	167.3655	0.00280	0.0000345	0.000029	0.0028	0.0055
86	94.8757	169.3761	94.8753	169.3757	0.00280	0.0000351	0.000029	0.0028	0.0055
87	96.8760	171.3868	96.8753	171.3861	0.00280	0.0000343	0.000029	0.0028	0.0055
88	98.8762	173.3974	98.8755	173.3967	0.00280	0.0000349	0.000029	0.0028	0.0055
89	100.8760	175.4076	100.8754	175.4070	0.00280	0.0000355	0.000029	0.0028	0.0055
90	102.8760	177.4180	102.8753	177.4173	0.00280	0.0000357	0.000029	0.0028	0.0055
91	104.8759	179.4283	104.8754	179.4278	0.00280	0.0000362	0.000029	0.0028	0.0055
92	106.8757	181.4385	106.8753	181.4381	0.00280	0.0000357	0.000029	0.0028	0.0055
93	108.8760	183.4492	108.8753	183.4485	0.00280	0.0000363	0.000029	0.0028	0.0055
94	110.8761	185.4597	110.8750	185.4586	0.00280	0.0000369	0.000029	0.0028	0.0055
95	112.8760	187.4700	112.8752	187.4692	0.00280	0.0000370	0.000029	0.0028	0.0055
96	114.8761	189.4805	114.8753	189.4797	0.00280	0.0000376	0.000029	0.0028	0.0055
97	116.8759	191.4907	116.8751	191.4899	0.00280	0.0000368	0.000029	0.0028	0.0055
98	118.8758	193.5010	118.8755	193.5007	0.00280	0.0000371	0.000029	0.0028	0.0055
99	120.8759	195.5115	120.8755	195.5111	0.00280	0.0000376	0.000029	0.0028	0.0055
100	122.8757	197.5217	122.8755	197.5215	0.00280	0.0000381	0.000029	0.0028	0.0055
101	124.8759	199.5323	124.8758	199.5322	0.00280	0.0000387	0.000029	0.0028	0.0055
102	126.8753	201.5421	126.8753	201.5421	0.00280	0.0000502	0.000029	0.0028	0.0055

The combined uncertainty (***u***_***c***_) is calculated from the adjusted uncertainty (***u***_***f***_), standard mass uncertainty (***u***_***s***_) and the uncertainty associated to the resolution of the analytical scale (***u***_***resolution***_) **–**this value can be obtained by dividing half the resolution of the instrument by the square root of three $\left(\mathrm{resolution}/2\sqrt{3}\right)$**,** to associate it with a rectangular probability distribution.

The coverture factor (***k***) is calculated form t-student distribution and a number of degrees of freedom φ=m−n, where ***m*** is a number of experimental data and ***n*** is the number of constant coefficients of the adjustment polynomial. For the specific case of the calibration of the analytical balance applying the Tare Function, the number of experimental points is equal to 204 and 2 are the constants of the first degree polynomial. Then, the calculated degree of freedom number is equal to 202.

Table 3 presented above confirms the value of the expanded uncertainty obtained (*U* = 0.0055 g) for a confidence level of 95.45%. This data confirms, despite being a low value compared to the resolution of the instrument, it can be improved by not using the tare function. That is, without affecting the electronic system of the balance. This is detailed in the next sections.

### 1.2 Calibration of the analytical scale without applying the tare function

Similarly, to the procedure described in the before section, the Table 4 presents the experimental data obtained when the Tare Function was not applied in the calibration analytical scale process. This table shows 204 experimental data and the standard mass value which was increasing each 0.5 g. The environmental temperature and the atmospheric pressure were measured for each experimental point.

**Table 4:** Experimental data (without applying the Tare Function).

Exp. Points	Standard Mass	Ascending load			Descending load		
		Mass indicated by the scale	Environmental Temperature	Atmospheric Pressure	Mass indicated by the scale	Environmental Temperature	Atmospheric Pressure
	g	g	°C	mbar/abs	g	°C	mbar/abs
1	0.0	0.0022	28.0	1002	0.0024	29.8	1000
2	0.5	0.5022	28.1	1002	0.5024	29.7	1000
3	2.5	2.5022	28.2	1002	2.5026	29.7	1000
4	4.5	4.5022	28.2	1002	4.5025	29.7	1000
5	6.5	6.5024	28.4	1002	6.5027	29.7	1000
6	8.5	8.5024	28.4	1002	8.5026	29.7	1000
7	10.5	10.5023	28.4	1002	10.5029	29.7	1000
8	12.5	12.5023	28.4	1002	12.5028	29.7	1000
9	14.5	14.5024	28.4	1002	14.5029	29.7	1000
10	16.5	16.5023	28.4	1002	16.5029	29.7	1000
11	18.5	18.5025	28.4	1002	18.5029	29.7	1000
12	20.5	20.5025	28.5	1002	20.5028	29.7	1000
13	22.5	22.5027	28.5	1002	22.5030	29.7	1000
14	24.5	24.5026	28.5	1002	24.5029	29.7	1000
15	26.5	26.5027	28.5	1002	26.5029	29.7	1000
16	28.5	28.5028	28.5	1002	28.5033	29.7	1000
17	30.5	30.5028	28.5	1002	30.5031	29.7	1000
18	32.5	32.5028	28.5	1002	32.5032	29.7	1000
19	34.5	34.5028	28.6	1002	34.5030	29.7	1000
20	36.5	36.5028	28.6	1002	36.5030	29.7	1000
21	38.5	38.5028	28.6	1002	38.5034	29.7	1000
22	40.5	40.5027	28.7	1002	40.5031	29.7	1000
23	42.5	42.5028	28.7	1002	42.5031	29.7	1000
24	44.5	44.5030	28.7	1002	44.5032	29.7	1000
25	46.5	46.5028	28.7	1002	46.5032	29.7	1000
26	48.5	48.5030	28.7	1002	48.5032	29.7	1000
27	50.5	50.5031	28.8	1002	50.5031	29.7	1000
28	52.5	52.5033	28.8	1001	52.5031	29.7	1000
29	54.5	54.5033	28.8	1001	54.5031	29.7	1000
30	56.5	56.5032	28.8	1001	56.5031	29.6	1000
31	58.5	58.5034	28.8	1001	58.5029	29.6	1000
32	60.5	60.5030	28.8	1001	60.5032	29.6	1000
33	62.5	62.5032	28.8	1001	62.5031	29.6	1000
34	64.5	64.5032	28.8	1001	64.5033	29.6	1000
35	66.5	66.5031	28.8	1001	66.5033	29.6	1000
36	68.5	68.5033	28.8	1001	68.5032	29.6	1000
37	70.5	70.5029	28.9	1001	70.5035	29.6	1000
38	72.5	72.5031	28.9	1001	72.5035	29.6	1000
39	74.5	74.5030	28.9	1001	74.5035	29.6	1000
40	76.5	76.5031	28.9	1001	76.5036	29.6	1000
41	78.5	78.5032	28.9	1001	78.5035	29.6	1000
42	80.5	80.5027	28.9	1001	80.5035	29.6	1000
43	82.5	82.5032	28.9	1001	82.5035	29.6	1000
44	84.5	84.5031	28.9	1001	84.5034	29.6	1000
45	86.5	86.5033	28.9	1001	86.5036	29.6	1000
46	88.5	88.5031	28.9	1001	88.5036	29.6	1000
47	90.5	90.5033	29.0	1001	90.5035	29.6	1000
48	92.5	92.5033	29.0	1001	92.5035	29.6	1000
49	94.5	94.5033	29.0	1001	94.5035	29.6	1000
50	96.5	96.5037	29.1	1001	96.5037	29.6	1000
51	98.5	98.5038	29.1	1001	98.5037	29.6	1000
52	100.5	100.5025	29.1	1001	100.5031	29.6	1000
53	102.5	102.5027	29.1	1001	102.5030	29.6	1000
54	104.5	104.5027	29.1	1001	104.5029	29.6	1000
55	106.5	106.5025	29.1	1001	106.5028	29.6	1000
56	108.5	108.5026	29.1	1001	108.5028	29.6	1000
57	110.5	110.5026	29.1	1001	110.5027	29.5	1000
58	112.5	112.5026	29.1	1001	112.5027	29.5	1000
59	114.5	114.5027	29.1	1001	114.5028	29.5	1000
60	116.5	116.5026	29.1	1001	116.5028	29.5	1000
61	118.5	118.5027	29.1	1001	118.5028	29.5	1000
62	120.5	120.5025	29.1	1001	120.5027	29.5	1000
63	122.5	122.5027	29.1	1001	122.5028	29.5	1000
64	124.5	124.5026	29.1	1001	124.5027	29.5	1000
65	126.5	126.5025	29.1	1001	126.5027	29.5	1000
66	128.5	128.5026	29.1	1001	128.5029	29.5	1000
67	130.5	130.5025	29.1	1001	130.5027	29.5	1000
68	132.5	132.5025	29.1	1001	132.5027	29.5	1000
69	134.5	134.5026	29.1	1001	134.5026	29.5	1000
70	136.5	136.5026	29.1	1001	136.5028	29.5	1000
71	138.5	138.5027	29.1	1001	138.5028	29.5	1000
72	140.5	140.5026	29.1	1001	140.5028	29.5	1000
73	142.5	142.5028	29.2	1001	142.5028	29.5	1000
74	144.5	144.5027	29.2	1001	144.5028	29.5	1000
75	146.5	146.5026	29.2	1001	146.5028	29.5	1000
76	148.5	148.5028	29.2	1001	148.5033	29.5	1000
77	150.5	150.5026	29.2	1001	150.5028	29.5	1000
78	152.5	152.5028	29.2	1001	152.5027	29.5	1000
79	154.5	154.5028	29.2	1000	154.5029	29.5	1000
80	156.5	156.5027	29.2	1000	156.5028	29.5	1000
81	158.5	158.5028	29.2	1000	158.5028	29.5	1000
82	160.5	160.5028	29.2	1000	160.5029	29.4	1000
83	162.5	162.5028	29.2	1000	162.5030	29.4	1000
84	164.5	164.5029	29.2	1000	164.5029	29.4	1000
85	166.5	166.5028	29.2	1000	166.5027	29.4	1000
86	168.5	168.5028	29.2	1000	168.5029	29.4	1000
87	170.5	170.5028	29.2	1000	170.5027	29.4	1000
88	172.5	172.5028	29.2	1000	172.5030	29.4	1000
89	174.5	174.5028	29.2	1000	174.5029	29.4	1000
90	176.5	176.5028	29.2	1000	176.5029	29.4	1000
91	178.5	178.5031	29.2	1000	178.5030	29.4	1000
92	180.5	180.5028	29.2	1000	180.5030	29.4	1000
93	182.5	182.5029	29.2	1000	182.5028	29.4	1000
94	184.5	184.5028	29.2	1000	184.5033	29.4	1000
95	186.5	186.5028	29.2	1000	186.5031	29.4	1000
96	188.5	188.5030	29.2	1000	188.5032	29.4	1000
97	190.5	190.5030	29.2	1000	190.5033	29.4	1000
98	192.5	192.5030	29.2	1000	192.5032	29.4	1000
99	194.5	194.5032	29.2	1000	194.5033	29.4	1000
100	196.5	196.5031	29.2	1000	196.5033	29.4	1000
101	198.5	198.5032	29.2	1000	198.5038	29.3	1000
102	200.5	200.5035	29.2	1000	200.5035	29.2	1000

For the ascending and descending load, Table 5 presents the experimental data for this new situation (without applying Tare Function), in terms of: indicated mass by the analytical scale, apparent mass calculated, uncertainty of apparent mass, environmental temperature, atmospheric pressure, air density, as well as its uncertainty.

**Fig. 2:**
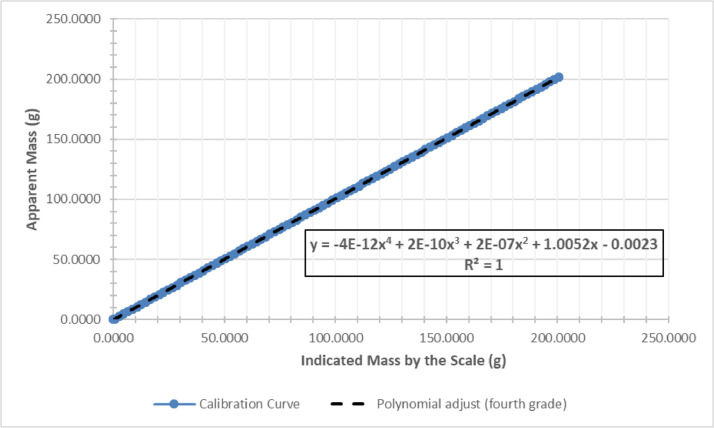
Polynomial adjustment curve (fourth degree polynomial).

Once the dataset showed in Table 5, the method of ordinary least squares was applied. This method allowed establishing a relationship between the mass indicated by the analytical scale (horizontal axis) and the apparent mass calculated (vertical axis). In this case, the polynomial that better adjusted the physical nature of the problem was a fourth degree polynomial and its uncertainty of adjustment (***u***_***f***_) is 0.00063 g. Fig. 2 shows the calibration curve obtained, as well as the adjustment equation. For this new situation, the mathematical model that best fits the physical phenomenon corresponds to a fourth degree polynomial whose constant coefficients are observed in Fig. 2. The determination coefficient confirms the excellent agreement between the mathematical and physical model.

Finally, Table 6 shows the dataset associated to expanded uncertainty. The corresponding coverage factor is equal to 1.97 (for a confidence level equal to 95.0%). However, in this case, the expanded uncertainty associated to mass measurement is less than the before situation. The estimated value was 0.0012 g.

**Table 6:** Uncertainty analysis (without applying the tare function).

Exp. Points	Ascending load		Descending load		Uncertainty analysis				
	Indicated Mass	Ajusted Mass	Indicated Mass	Ajusted Mass	Adjusted Uncertainty	Standard Mass Uncertainty	Resolution Uncertainty	Combined Uncertainty	Expanded Uncertainty
	g	G	g	g	g	g	g	g	g
1	0.0022	−0.0007	0.0024	74.0094	0.00063	0.0000000	0.000029	0.00063	0.0012
2	0.5022	0.5019	0.5024	74.5120	0.00063	0.0000042	0.000029	0.00063	0.0012
3	2.5022	2.5123	2.5026	76.5226	0.00063	0.0000077	0.000029	0.00063	0.0012
4	4.5022	4.5227	4.5025	78.5329	0.00063	0.0000101	0.000029	0.00063	0.0012
5	6.5024	6.5333	6.5027	80.5435	0.00063	0.0000107	0.000029	0.00063	0.0012
6	8.5024	8.5437	8.5026	82.5538	0.00063	0.0000125	0.000029	0.00063	0.0012
7	10.5023	10.5540	10.5029	84.5645	0.00063	0.0000108	0.000029	0.00063	0.0012
8	12.5023	12.5644	12.5028	86.5748	0.00063	0.0000126	0.000029	0.00063	0.0012
9	14.5024	14.5749	14.5029	88.5853	0.00063	0.0000142	0.000029	0.00063	0.0012
10	16.5023	16.5852	16.5029	90.5957	0.00063	0.0000146	0.000029	0.00063	0.0012
11	18.5025	18.5958	18.5029	92.6061	0.00063	0.0000160	0.000029	0.00063	0.0012
12	20.5025	20.6062	20.5028	94.6164	0.00063	0.0000141	0.000029	0.00063	0.0012
13	22.5027	22.6168	22.5030	96.6270	0.00063	0.0000155	0.000029	0.00063	0.0012
14	24.5026	24.6271	24.5029	98.6373	0.00063	0.0000169	0.000029	0.00063	0.0012
15	26.5027	26.6376	26.5029	100.6477	0.00063	0.0000167	0.000029	0.00063	0.0012
16	28.5028	28.6481	28.5033	102.6585	0.00063	0.0000179	0.000029	0.00063	0.0012
17	30.5028	30.6585	30.5031	104.6687	0.00063	0.0000173	0.000029	0.00063	0.0012
18	32.5028	32.6689	32.5032	106.6792	0.00063	0.0000185	0.000029	0.00063	0.0012
19	34.5028	34.6793	34.5030	108.6894	0.00063	0.0000196	0.000029	0.00063	0.0012
20	36.5028	36.6897	36.5030	110.6998	0.00063	0.0000199	0.000029	0.00063	0.0012
21	38.5028	38.7001	38.5034	112.7106	0.00063	0.0000210	0.000029	0.00063	0.0012
22	40.5027	40.7104	40.5031	114.7207	0.00063	0.0000195	0.000029	0.00063	0.0012
23	42.5028	42.7209	42.5031	116.7311	0.00063	0.0000206	0.000029	0.00063	0.0012
24	44.5030	44.7315	44.5032	118.7416	0.00063	0.0000216	0.000029	0.00063	0.0012
25	46.5028	46.7417	46.5032	120.7520	0.00063	0.0000219	0.000029	0.00063	0.0012
26	48.5030	48.7523	48.5032	122.7624	0.00063	0.0000228	0.000029	0.00063	0.0012
27	50.5031	50.7628	50.5031	124.7727	0.00063	0.0000170	0.000029	0.00063	0.0012
28	52.5033	52.7734	52.5031	126.7831	0.00063	0.0000182	0.000029	0.00063	0.0012
29	54.5033	54.7838	54.5031	128.7935	0.00063	0.0000193	0.000029	0.00063	0.0012
30	56.5032	56.7941	56.5031	130.8039	0.00063	0.0000197	0.000029	0.00063	0.0012
31	58.5034	58.8047	58.5029	132.8141	0.00063	0.0000207	0.000029	0.00063	0.0012
32	60.5030	60.8147	60.5032	134.8248	0.00063	0.0000197	0.000029	0.00063	0.0012
33	62.5032	62.8253	62.5031	136.8351	0.00063	0.0000208	0.000029	0.00063	0.0012
34	64.5032	64.8357	64.5033	138.8457	0.00063	0.0000218	0.000029	0.00063	0.0012
35	66.5031	66.8460	66.5033	140.8561	0.00063	0.0000221	0.000029	0.00063	0.0012
36	68.5033	68.8566	68.5032	142.8664	0.00063	0.0000230	0.000029	0.00063	0.0012
37	70.5029	70.8666	70.5035	144.8771	0.00063	0.0000217	0.000029	0.00063	0.0012
38	72.5031	72.8772	72.5035	146.8875	0.00063	0.0000227	0.000029	0.00063	0.0012
39	74.5030	74.8875	74.5035	148.8979	0.00063	0.0000236	0.000029	0.00063	0.0012
40	76.5031	76.8980	76.5036	150.9084	0.00063	0.0000239	0.000029	0.00063	0.0012
41	78.5032	78.9085	78.5035	152.9187	0.00063	0.0000247	0.000029	0.00063	0.0012
42	80.5027	80.9184	80.5035	154.9291	0.00063	0.0000239	0.000029	0.00063	0.0012
43	82.5032	82.9293	82.5035	156.9395	0.00063	0.0000248	0.000029	0.00063	0.0012
44	84.5031	84.9396	84.5034	158.9498	0.00063	0.0000256	0.000029	0.00063	0.0012
45	86.5033	86.9502	86.5036	160.9604	0.00063	0.0000239	0.000029	0.00063	0.0012
46	88.5031	88.9604	88.5036	162.9708	0.00063	0.0000247	0.000029	0.00063	0.0012
47	90.5033	90.9710	90.5035	164.9811	0.00063	0.0000256	0.000029	0.00063	0.0012
48	92.5033	92.9814	92.5035	166.9915	0.00063	0.0000264	0.000029	0.00063	0.0012
49	94.5033	94.9918	94.5035	169.0019	0.00063	0.0000272	0.000029	0.00063	0.0012
50	96.5037	97.0026	96.5037	171.0125	0.00063	0.0000274	0.000029	0.00063	0.0012
51	98.5038	99.0131	98.5037	173.0229	0.00063	0.0000282	0.000029	0.00063	0.0012
52	100.5025	101.0222	100.5031	175.0327	0.00063	0.0000268	0.000029	0.00063	0.0012
53	102.5027	103.0328	102.5030	177.0430	0.00063	0.0000276	0.000029	0.00063	0.0012
54	104.5027	105.0432	104.5029	179.0533	0.00063	0.0000284	0.000029	0.00063	0.0012
55	106.5025	107.0534	106.5028	181.0636	0.00063	0.0000286	0.000029	0.00063	0.0012
56	108.5026	109.0639	108.5028	183.0740	0.00063	0.0000293	0.000029	0.00063	0.0012
57	110.5026	111.0743	110.5027	185.0843	0.00063	0.0000286	0.000029	0.00063	0.0012
58	112.5026	113.0847	112.5027	187.0947	0.00063	0.0000294	0.000029	0.00063	0.0012
59	114.5027	115.0952	114.5028	189.1052	0.00063	0.0000301	0.000029	0.00063	0.0012
60	116.5026	117.1055	116.5028	191.1156	0.00063	0.0000303	0.000029	0.00063	0.0012
61	118.5027	119.1160	118.5028	193.1260	0.00063	0.0000310	0.000029	0.00063	0.0012
62	120.5025	121.1262	120.5027	195.1363	0.00063	0.0000300	0.000029	0.00063	0.0012
63	122.5027	123.1368	122.5028	197.1468	0.00063	0.0000307	0.000029	0.00063	0.0012
64	124.5026	125.1471	124.5027	199.1571	0.00063	0.0000314	0.000029	0.00063	0.0012
65	126.5025	127.1574	126.5027	201.1675	0.00063	0.0000316	0.000029	0.00063	0.0012
66	128.5026	129.1679	128.5029	203.1781	0.00063	0.0000323	0.000029	0.00063	0.0012
67	130.5025	131.1782	130.5027	205.1883	0.00063	0.0000317	0.000029	0.00063	0.0012
68	132.5025	133.1886	132.5027	207.1987	0.00063	0.0000323	0.000029	0.00063	0.0012
69	134.5026	135.1991	134.5026	209.2090	0.00063	0.0000330	0.000029	0.00063	0.0012
70	136.5026	137.2095	136.5028	211.2196	0.00063	0.0000332	0.000029	0.00063	0.0012
71	138.5027	139.2200	138.5028	213.2300	0.00063	0.0000338	0.000029	0.00063	0.0012
72	140.5026	141.2303	140.5028	215.2404	0.00063	0.0000329	0.000029	0.00063	0.0012
73	142.5028	143.2409	142.5028	217.2508	0.00063	0.0000336	0.000029	0.00063	0.0012
74	144.5027	145.2512	144.5028	219.2612	0.00063	0.0000342	0.000029	0.00063	0.0012
75	146.5026	147.2615	146.5028	221.2716	0.00063	0.0000344	0.000029	0.00063	0.0012
76	148.5028	149.2721	148.5033	223.2825	0.00063	0.0000350	0.000029	0.00063	0.0012
77	150.5026	151.2823	150.5028	225.2924	0.00063	0.0000315	0.000029	0.00063	0.0012
78	152.5028	153.2929	152.5027	227.3027	0.00063	0.0000322	0.000029	0.00063	0.0012
79	154.5028	155.3033	154.5029	229.3133	0.00063	0.0000328	0.000029	0.00063	0.0012
80	156.5027	157.3136	156.5028	231.3236	0.00063	0.0000330	0.000029	0.00063	0.0012
81	158.5028	159.3241	158.5028	233.3340	0.00063	0.0000336	0.000029	0.00063	0.0012
82	160.5028	161.3345	160.5029	235.3445	0.00063	0.0000330	0.000029	0.00063	0.0012
83	162.5028	163.3449	162.5030	237.3550	0.00063	0.0000337	0.000029	0.00063	0.0012
84	164.5029	165.3554	164.5029	239.3653	0.00063	0.0000343	0.000029	0.00063	0.0012
85	166.5028	167.3657	166.5027	241.3755	0.00063	0.0000345	0.000029	0.00063	0.0012
86	168.5028	169.3761	168.5029	243.3861	0.00063	0.0000351	0.000029	0.00063	0.0012
87	170.5028	171.3865	170.5027	245.3963	0.00063	0.0000343	0.000029	0.00063	0.0012
88	172.5028	173.3969	172.5030	247.4070	0.00063	0.0000349	0.000029	0.00063	0.0012
89	174.5028	175.4073	174.5029	249.4173	0.00063	0.0000355	0.000029	0.00063	0.0012
90	176.5028	177.4177	176.5029	251.4277	0.00063	0.0000357	0.000029	0.00063	0.0012
91	178.5031	179.4284	178.5030	253.4382	0.00063	0.0000362	0.000029	0.00063	0.0012
92	180.5028	181.4385	180.5030	255.4486	0.00063	0.0000357	0.000029	0.00063	0.0012
93	182.5029	183.4490	182.5028	257.4588	0.00063	0.0000363	0.000029	0.00063	0.0012
94	184.5028	185.4593	184.5033	259.4697	0.00063	0.0000369	0.000029	0.00063	0.0012
95	186.5028	187.4697	186.5031	261.4799	0.00063	0.0000370	0.000029	0.00063	0.0012
96	188.5030	189.4803	188.5032	263.4904	0.00063	0.0000376	0.000029	0.00063	0.0012
97	190.5030	191.4907	190.5033	265.5009	0.00063	0.0000368	0.000029	0.00063	0.0012
98	192.5030	193.5011	192.5032	267.5112	0.00063	0.0000371	0.000029	0.00063	0.0012
99	194.5032	195.5117	194.5033	269.5217	0.00063	0.0000376	0.000029	0.00063	0.0012
100	196.5031	197.5220	196.5033	271.5321	0.00063	0.0000381	0.000029	0.00063	0.0012
101	198.5032	199.5325	198.5038	273.5430	0.00063	0.0000387	0.000029	0.00063	0.0012
102	200.5035	201.5432	200.5035	275.5531	0.00063	0.0000502	0.000029	0.00063	0.0012

According to the data presented in Table 6 above, it is observed that the expanded uncertainty (*U* = 0.0012 g) is approximately five times less than that presented in Table 3 (*U* = 0.0055 g), which confirms that there is an undesirable measurement error that contributes to a dismissal of the metrological reliability of a digital scale when using the tare function.

## 2 Experimental design, materials and methods

**Fig. 3:**
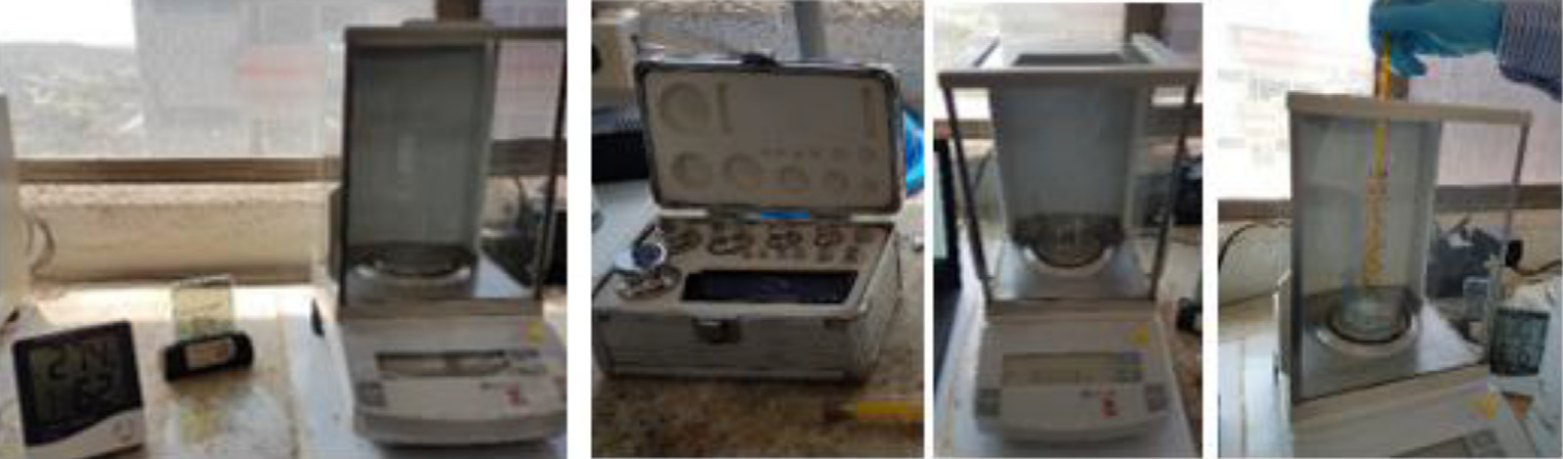
Experimental assembly scheme.

In the course of the experiments, carried out in a chemical laboratory [5], an analytical balance (manufacturer: Ohaus) with a resolution of 0.0001 g and a measurement range from 0 to 210 g was used for mass measurement. The graduated cylinder used (manufacturer: Brand) has a volumetric capacity declared in 50 ml and a scale division equal to 1 ml. For the measurement of liquid temperature, a liquid in glass thermometer with a measuring range of 0 to 100 °C and a scale division equal to 0.1 °C was used. For the measurement of the environmental conditions a digital thermohygrometer (manufacturer: HTC) was used with a resolution equal to 0.1 °C and a measuring range of 0.0–99.9 °C in temperature. In addition, to offering a resolution of 1% RH and a range maximum up to 80% RH for the case of relative humidity (RH) measurement. Additionally, a barometer with a resolution of 1 mbar was used to measure the atmospheric pressure. Fig. 3 illustrates an experimental assembly scheme.

The experiment was started by calibrating the analytical balance according to the OIML R-76–1 guidelines. An E1 class weight set [5,10] was used. Two situations were evaluated in the calibration of the graduated cylinder: (i) placing the graduated cylinder in the load cell and applying the tare function. Subsequently, the graduated cylinder is removed from the load cell and the calibration process begins; (ii) initiating the calibration process without discounting the mass of the graduated cylinder. In both cases, once the scale was switched on, there was a 90-minute wait for the stabilization of the electronic system, according to the recommendations of the manufacturer. In addition, in both situations evaluated, 204 experimental points were obtained (102 in for Ascending Load and 102 for Descending Load.). Once the first calibration of the balance was completed (Applying the Tare Function), the graduated cylinder was calibrated. Pure water was used for this activity [11] and the total volume of the graduated cylinder was achieved by introducing small volumes (approximately 0.5 ml in 0.5 ml). At each experimental point the water temperature was measured with the help of the liquid glass thermometer. Additionally, atmospheric pressure and ambient temperature were measured (manufacturer: Forecast; Range: 0 a 9999 mbar; Resolution: 1 mbar). The objective of increasing the volume of the graduated cylinder in small volumes was to reduce the impact of non-homogeneity related to the internal diameter.

**Fig. 4:**
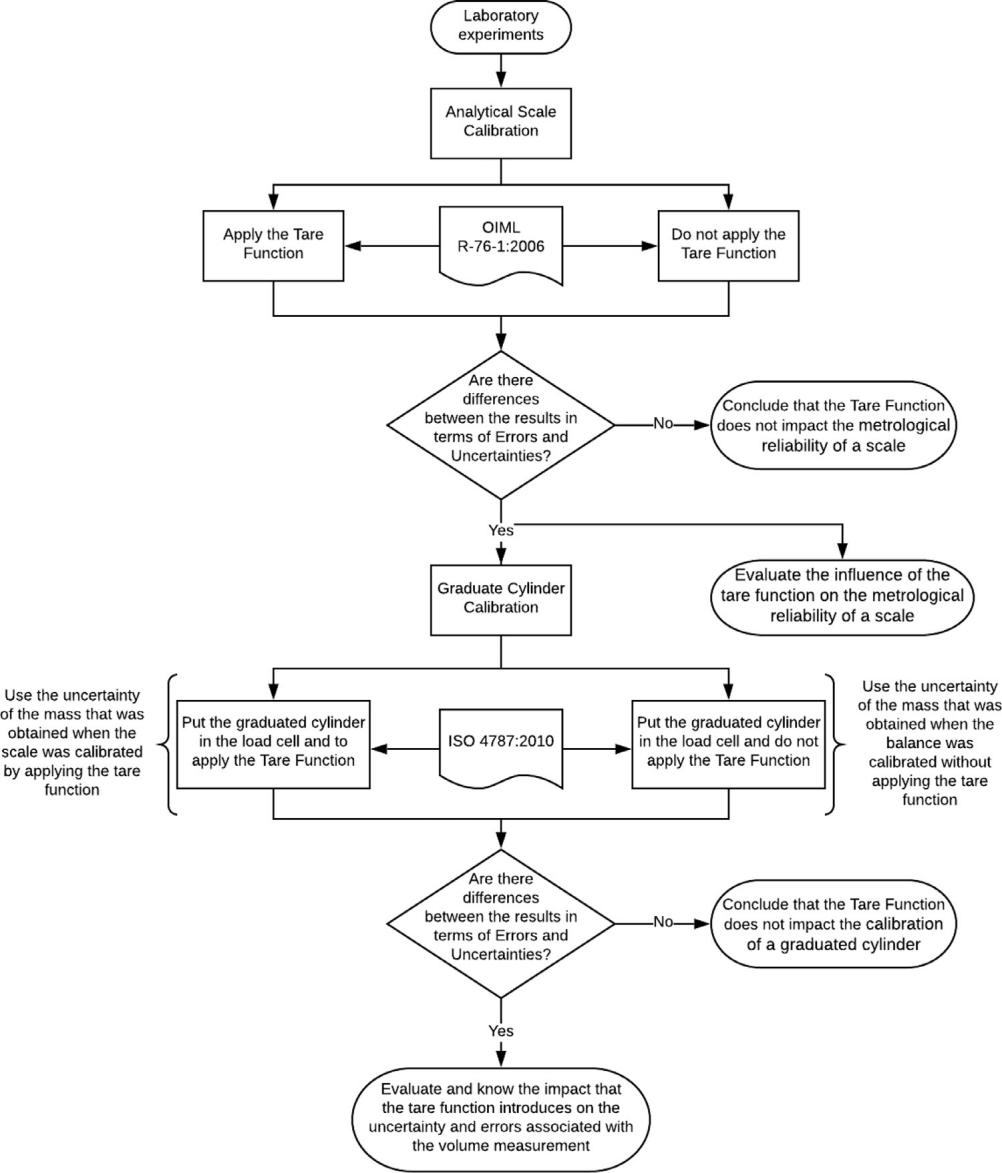
Flowchart of to the experimental methodology.

At the end of this process, the scale calibration started again, however, in this situation, the tare function was not applied. Once the calibration of the balance was finished, the calibration of the graduated cylinder was carried out without discounting its initial mass (that is, without applying the tare function). The calibration procedure of the graduated cylinder followed the one described above. In both cases where the graduated cylinder was calibrated, 91 experimental points were obtained, to total the maximum volumetric capacity of 50 ml. Fig. 4 illustrates a flow chart related to the methodology adopted in the research carried out.

## Declaration of Competing Interest

The authors declare that they have no known competing financial interests or personal relationships which have, or could be perceived to have, influenced the work reported in this article.

## Appendix Supplementary materials

Image, application 1

Image, application 2

Image, application 3
