# Supplementary material for: Evaluation of the metrological reliability of a graduated cylinder from experimental data from an in-situ calibration
Source: Data Brief. 2020 Aug 6;32:106133. doi: 10.1016/j.dib.2020.106133 (PMC7406472; doi:10.1016/j.dib.2020.106133)
Supplement: Supplementary file 2 [file mmc2.docx]

**Table 2** – Experimental Data (applying the Tare Function)

| **Exp. Point** | **Standard Mass** | | | **ASCENDING LOAD** | | | | | | | | | | | | | **DESCENDING LOAD** | | | | | | | | | | | |
| --- | --- | --- | --- | --- | --- | --- | --- | --- | --- | --- | --- | --- | --- | --- | --- | --- | --- | --- | --- | --- | --- | --- | --- | --- | --- | --- | --- | --- |
|  |  |  |  | **Analytical Scale** | | | | **Environmental Temperature** | | | **Atmospheric Pressure** | | | **Air Density** | | | **Analytical Scale** | | | | **Environmental Temperature** | | | **Atmospheric Pressure** | | | **Air Density** | |
|  | **Mass** | **Uncertainty** | **Indicated Mass** | | **Apparent Mass** | **Uncertainty of Apparent Mass** | **Indicated Temperature** | | **Uncertainty** | **Indicated Pressure** | | **Uncertainty** | **Air density calculated** | | **Uncertainty** | **Indicated Mass** | | **Apparent Mass** | **Uncertainty of Apparent Mass** | **Indicated Temperature** | | **Uncertainty** | **Indicated Pressure** | | **Uncertainty** | **Air density calculated** | | **Uncertainty** |
|  | **g** | **g** | **g** | | **g** | **g** | **^o^C** | | **^o^C** | **mbar/abs** | | **mbar/abs** | **kg/m^3^** | | **kg/m^3^** | **g** | | **g** | **g** | **^o^C** | | **^o^C** | **mbar/abs** | | **mbar/abs** | **kg/m^3^** | | **kg/m^3^** |
| 1 | 0.0 | 0.0000000 | -73.6239 | | 0.0000 | 0.000000 | 32.8 | | 0.029 | 1001 | | 0.60 | 1.14 | | 0.00069 | -73.6145 | | 0.0000 | 0.000000 | 29.8 | | 0.029 | 1000 | | 0.60 | 1.15 | | 0.00070 |
| 2 | 0.5 | 0.0000042 | -73.1240 | | 0.5026 | 0.000004 | 32.8 | | 0.029 | 1001 | | 0.60 | 1.14 | | 0.00069 | -73.1155 | | 0.5026 | 0.000004 | 30.4 | | 0.029 | 1002 | | 0.60 | 1.15 | | 0.00070 |
| 3 | 2.5 | 0.0000077 | -71.1238 | | 2.5130 | 0.000008 | 32.5 | | 0.029 | 1001 | | 0.60 | 1.14 | | 0.00069 | -71.1165 | | 2.5130 | 0.000008 | 30.4 | | 0.029 | 1002 | | 0.60 | 1.15 | | 0.00070 |
| 4 | 4.5 | 0.0000101 | -69.1240 | | 4.5235 | 0.000010 | 32.0 | | 0.029 | 1001 | | 0.60 | 1.14 | | 0.00069 | -69.1166 | | 4.5235 | 0.000010 | 30.4 | | 0.029 | 1002 | | 0.60 | 1.15 | | 0.00070 |
| 5 | 6.5 | 0.0000107 | -67.1233 | | 6.5339 | 0.000011 | 31.7 | | 0.029 | 1001 | | 0.60 | 1.14 | | 0.00069 | -67.1175 | | 6.5339 | 0.000011 | 30.4 | | 0.029 | 1002 | | 0.60 | 1.15 | | 0.00070 |
| 6 | 8.5 | 0.0000125 | -65.1234 | | 8.5444 | 0.000013 | 31.5 | | 0.029 | 1001 | | 0.60 | 1.14 | | 0.00069 | -65.1179 | | 8.5444 | 0.000013 | 30.4 | | 0.029 | 1002 | | 0.60 | 1.15 | | 0.00070 |
| 7 | 10.5 | 0.0000108 | -63.1233 | | 10.5548 | 0.000011 | 31.4 | | 0.029 | 1002 | | 0.60 | 1.15 | | 0.00069 | -63.1175 | | 10.5548 | 0.000011 | 30.4 | | 0.029 | 1002 | | 0.60 | 1.15 | | 0.00070 |
| 8 | 12.5 | 0.0000126 | -61.1235 | | 12.5652 | 0.000013 | 31.3 | | 0.029 | 1001 | | 0.60 | 1.15 | | 0.00070 | -61.1182 | | 12.5652 | 0.000013 | 30.4 | | 0.029 | 1002 | | 0.60 | 1.15 | | 0.00070 |
| 9 | 14.5 | 0.0000142 | -59.1237 | | 14.5757 | 0.000014 | 31.3 | | 0.029 | 1002 | | 0.60 | 1.15 | | 0.00070 | -59.1178 | | 14.5757 | 0.000014 | 30.4 | | 0.029 | 1002 | | 0.60 | 1.15 | | 0.00070 |
| 10 | 16.5 | 0.0000146 | -57.1233 | | 16.5861 | 0.000015 | 31.2 | | 0.029 | 1002 | | 0.60 | 1.15 | | 0.00070 | -57.1189 | | 16.5861 | 0.000015 | 30.4 | | 0.029 | 1002 | | 0.60 | 1.15 | | 0.00070 |
| 11 | 18.5 | 0.0000160 | -55.1236 | | 18.5966 | 0.000016 | 31.2 | | 0.029 | 1002 | | 0.60 | 1.15 | | 0.00070 | -55.1192 | | 18.5966 | 0.000016 | 30.4 | | 0.029 | 1002 | | 0.60 | 1.15 | | 0.00070 |
| 12 | 20.5 | 0.0000141 | -53.1231 | | 20.6070 | 0.000014 | 31.2 | | 0.029 | 1002 | | 0.60 | 1.15 | | 0.00070 | -53.1204 | | 20.6070 | 0.000014 | 30.4 | | 0.029 | 1001 | | 0.60 | 1.15 | | 0.00070 |
| 13 | 22.5 | 0.0000155 | -51.1234 | | 22.6174 | 0.000016 | 31.1 | | 0.029 | 1002 | | 0.60 | 1.15 | | 0.00070 | -51.1199 | | 22.6174 | 0.000016 | 30.4 | | 0.029 | 1001 | | 0.60 | 1.15 | | 0.00070 |
| 14 | 24.5 | 0.0000169 | -49.1232 | | 24.6279 | 0.000017 | 31.1 | | 0.029 | 1002 | | 0.60 | 1.15 | | 0.00070 | -49.1206 | | 24.6279 | 0.000017 | 30.4 | | 0.029 | 1001 | | 0.60 | 1.15 | | 0.00070 |
| 15 | 26.5 | 0.0000167 | -47.1232 | | 26.6383 | 0.000017 | 31.1 | | 0.029 | 1002 | | 0.60 | 1.15 | | 0.00070 | -47.1210 | | 26.6383 | 0.000017 | 30.4 | | 0.029 | 1001 | | 0.60 | 1.15 | | 0.00070 |
| 16 | 28.5 | 0.0000179 | -45.1233 | | 28.6488 | 0.000018 | 31.1 | | 0.029 | 1002 | | 0.60 | 1.15 | | 0.00070 | -45.1212 | | 28.6488 | 0.000018 | 30.4 | | 0.029 | 1001 | | 0.60 | 1.15 | | 0.00070 |
| 17 | 30.5 | 0.0000173 | -43.1233 | | 30.6592 | 0.000018 | 31.0 | | 0.029 | 1002 | | 0.60 | 1.15 | | 0.00070 | -43.1210 | | 30.6592 | 0.000018 | 30.4 | | 0.029 | 1001 | | 0.60 | 1.15 | | 0.00070 |
| 18 | 32.5 | 0.0000185 | -41.1235 | | 32.6696 | 0.000019 | 31.0 | | 0.029 | 1002 | | 0.60 | 1.15 | | 0.00070 | -41.1215 | | 32.6696 | 0.000019 | 30.4 | | 0.029 | 1001 | | 0.60 | 1.15 | | 0.00070 |
| 19 | 34.5 | 0.0000196 | -39.1233 | | 34.6801 | 0.000020 | 30.9 | | 0.029 | 1002 | | 0.60 | 1.15 | | 0.00070 | -39.1229 | | 34.6801 | 0.000020 | 30.4 | | 0.029 | 1001 | | 0.60 | 1.15 | | 0.00070 |
| 20 | 36.5 | 0.0000199 | -37.1235 | | 36.6905 | 0.000020 | 30.9 | | 0.029 | 1002 | | 0.60 | 1.15 | | 0.00070 | -37.1227 | | 36.6905 | 0.000020 | 30.4 | | 0.029 | 1001 | | 0.60 | 1.15 | | 0.00070 |
| 21 | 38.5 | 0.0000210 | -35.1232 | | 38.7010 | 0.000021 | 30.9 | | 0.029 | 1002 | | 0.60 | 1.15 | | 0.00070 | -35.1223 | | 38.7010 | 0.000021 | 30.4 | | 0.029 | 1001 | | 0.60 | 1.15 | | 0.00070 |
| 22 | 40.5 | 0.0000195 | -33.1217 | | 40.7114 | 0.000020 | 30.9 | | 0.029 | 1002 | | 0.60 | 1.15 | | 0.00070 | -33.1230 | | 40.7114 | 0.000020 | 30.4 | | 0.029 | 1001 | | 0.60 | 1.15 | | 0.00070 |
| 23 | 42.5 | 0.0000206 | -31.1218 | | 42.7218 | 0.000021 | 30.8 | | 0.029 | 1002 | | 0.60 | 1.15 | | 0.00070 | -31.1220 | | 42.7218 | 0.000021 | 30.4 | | 0.029 | 1001 | | 0.60 | 1.15 | | 0.00070 |
| 24 | 44.5 | 0.0000216 | -29.1215 | | 44.7323 | 0.000022 | 30.8 | | 0.029 | 1002 | | 0.60 | 1.15 | | 0.00070 | -29.1229 | | 44.7323 | 0.000022 | 30.4 | | 0.029 | 1001 | | 0.60 | 1.15 | | 0.00070 |
| 25 | 46.5 | 0.0000219 | -27.1216 | | 46.7427 | 0.000022 | 30.8 | | 0.029 | 1002 | | 0.60 | 1.15 | | 0.00070 | -27.1229 | | 46.7427 | 0.000022 | 30.4 | | 0.029 | 1001 | | 0.60 | 1.15 | | 0.00070 |
| 26 | 48.5 | 0.0000228 | -25.1216 | | 48.7532 | 0.000023 | 30.8 | | 0.029 | 1002 | | 0.60 | 1.15 | | 0.00070 | -25.1230 | | 48.7532 | 0.000023 | 30.4 | | 0.029 | 1001 | | 0.60 | 1.15 | | 0.00070 |
| 27 | 50.5 | 0.0000170 | -23.1216 | | 50.7636 | 0.000018 | 30.7 | | 0.029 | 1002 | | 0.60 | 1.15 | | 0.00070 | -23.1230 | | 50.7636 | 0.000018 | 30.4 | | 0.029 | 1001 | | 0.60 | 1.15 | | 0.00070 |
| 28 | 52.5 | 0.0000182 | -21.1217 | | 52.7740 | 0.000019 | 30.7 | | 0.029 | 1002 | | 0.60 | 1.15 | | 0.00070 | -21.1228 | | 52.7740 | 0.000019 | 30.4 | | 0.029 | 1001 | | 0.60 | 1.15 | | 0.00070 |
| 29 | 54.5 | 0.0000193 | -19.1219 | | 54.7845 | 0.000020 | 30.7 | | 0.029 | 1002 | | 0.60 | 1.15 | | 0.00070 | -19.1232 | | 54.7845 | 0.000020 | 30.4 | | 0.029 | 1001 | | 0.60 | 1.15 | | 0.00070 |
| 30 | 56.5 | 0.0000197 | -17.1218 | | 56.7949 | 0.000020 | 30.6 | | 0.029 | 1002 | | 0.60 | 1.15 | | 0.00070 | -17.1229 | | 56.7949 | 0.000020 | 30.4 | | 0.029 | 1001 | | 0.60 | 1.15 | | 0.00070 |
| 31 | 58.5 | 0.0000207 | -15.1218 | | 58.8053 | 0.000021 | 30.6 | | 0.029 | 1002 | | 0.60 | 1.15 | | 0.00070 | -15.1227 | | 58.8053 | 0.000021 | 30.4 | | 0.029 | 1001 | | 0.60 | 1.15 | | 0.00070 |
| 32 | 60.5 | 0.0000197 | -13.1218 | | 60.8158 | 0.000021 | 30.5 | | 0.029 | 1002 | | 0.60 | 1.15 | | 0.00070 | -13.1230 | | 60.8158 | 0.000021 | 30.4 | | 0.029 | 1001 | | 0.60 | 1.15 | | 0.00070 |
| 33 | 62.5 | 0.0000208 | -11.1219 | | 62.8262 | 0.000022 | 30.5 | | 0.029 | 1002 | | 0.60 | 1.15 | | 0.00070 | -11.1230 | | 62.8262 | 0.000022 | 30.4 | | 0.029 | 1001 | | 0.60 | 1.15 | | 0.00070 |
| 34 | 64.5 | 0.0000218 | -9.1216 | | 64.8367 | 0.000023 | 30.5 | | 0.029 | 1002 | | 0.60 | 1.15 | | 0.00070 | -9.1229 | | 64.8367 | 0.000023 | 30.4 | | 0.029 | 1001 | | 0.60 | 1.15 | | 0.00070 |
| 35 | 66.5 | 0.0000221 | -7.1220 | | 66.8471 | 0.000023 | 30.5 | | 0.029 | 1002 | | 0.60 | 1.15 | | 0.00070 | -7.1229 | | 66.8471 | 0.000023 | 30.4 | | 0.029 | 1001 | | 0.60 | 1.15 | | 0.00070 |
| 36 | 68.5 | 0.0000230 | -5.1220 | | 68.8575 | 0.000024 | 30.5 | | 0.029 | 1002 | | 0.60 | 1.15 | | 0.00070 | -5.1230 | | 68.8575 | 0.000024 | 30.4 | | 0.029 | 1001 | | 0.60 | 1.15 | | 0.00070 |
| 37 | 70.5 | 0.0000217 | -3.1223 | | 70.8680 | 0.000023 | 30.5 | | 0.029 | 1002 | | 0.60 | 1.15 | | 0.00070 | -3.1229 | | 70.8680 | 0.000023 | 30.4 | | 0.029 | 1001 | | 0.60 | 1.15 | | 0.00070 |
| 38 | 72.5 | 0.0000227 | -1.1220 | | 72.8784 | 0.000024 | 30.5 | | 0.029 | 1002 | | 0.60 | 1.15 | | 0.00070 | -1.1228 | | 72.8784 | 0.000024 | 30.4 | | 0.029 | 1001 | | 0.60 | 1.15 | | 0.00070 |
| 39 | 74.5 | 0.0000236 | 0.8780 | | 74.8889 | 0.000025 | 30.4 | | 0.029 | 1002 | | 0.60 | 1.15 | | 0.00070 | 0.8772 | | 74.8889 | 0.000025 | 30.4 | | 0.029 | 1001 | | 0.60 | 1.15 | | 0.00070 |
| 40 | 76.5 | 0.0000239 | 2.8777 | | 76.8993 | 0.000025 | 30.4 | | 0.029 | 1002 | | 0.60 | 1.15 | | 0.00070 | 2.8770 | | 76.8993 | 0.000025 | 30.4 | | 0.029 | 1001 | | 0.60 | 1.15 | | 0.00070 |
| 41 | 78.5 | 0.0000247 | 4.8775 | | 78.9097 | 0.000026 | 30.4 | | 0.029 | 1002 | | 0.60 | 1.15 | | 0.00070 | 4.8773 | | 78.9097 | 0.000026 | 30.4 | | 0.029 | 1001 | | 0.60 | 1.15 | | 0.00070 |
| 42 | 80.5 | 0.0000239 | 6.8772 | | 80.9202 | 0.000025 | 30.4 | | 0.029 | 1002 | | 0.60 | 1.15 | | 0.00070 | 6.8771 | | 80.9202 | 0.000025 | 30.4 | | 0.029 | 1001 | | 0.60 | 1.15 | | 0.00070 |
| 43 | 82.5 | 0.0000248 | 8.8773 | | 82.9306 | 0.000026 | 30.4 | | 0.029 | 1002 | | 0.60 | 1.15 | | 0.00070 | 8.8769 | | 82.9306 | 0.000026 | 30.4 | | 0.029 | 1001 | | 0.60 | 1.15 | | 0.00070 |
| 44 | 84.5 | 0.0000256 | 10.8774 | | 84.9411 | 0.000027 | 30.4 | | 0.029 | 1002 | | 0.60 | 1.15 | | 0.00070 | 10.8773 | | 84.9411 | 0.000027 | 30.4 | | 0.029 | 1001 | | 0.60 | 1.15 | | 0.00070 |
| 45 | 86.5 | 0.0000239 | 12.8778 | | 86.9515 | 0.000025 | 30.4 | | 0.029 | 1002 | | 0.60 | 1.15 | | 0.00070 | 12.8770 | | 86.9515 | 0.000025 | 30.4 | | 0.029 | 1002 | | 0.60 | 1.15 | | 0.00070 |
| 46 | 88.5 | 0.0000247 | 14.8776 | | 88.9619 | 0.000026 | 30.4 | | 0.029 | 1002 | | 0.60 | 1.15 | | 0.00070 | 14.8766 | | 88.9619 | 0.000026 | 30.4 | | 0.029 | 1002 | | 0.60 | 1.15 | | 0.00070 |
| 47 | 90.5 | 0.0000256 | 16.8777 | | 90.9724 | 0.000027 | 30.4 | | 0.029 | 1002 | | 0.60 | 1.15 | | 0.00070 | 16.8770 | | 90.9724 | 0.000027 | 30.4 | | 0.029 | 1002 | | 0.60 | 1.15 | | 0.00070 |
| 48 | 92.5 | 0.0000264 | 18.8777 | | 92.9828 | 0.000028 | 30.4 | | 0.029 | 1002 | | 0.60 | 1.15 | | 0.00070 | 18.8770 | | 92.9828 | 0.000028 | 30.4 | | 0.029 | 1002 | | 0.60 | 1.15 | | 0.00070 |
| 49 | 94.5 | 0.0000272 | 20.8779 | | 94.9933 | 0.000029 | 30.4 | | 0.029 | 1002 | | 0.60 | 1.15 | | 0.00070 | 20.8768 | | 94.9933 | 0.000029 | 30.4 | | 0.029 | 1002 | | 0.60 | 1.15 | | 0.00070 |
| 50 | 96.5 | 0.0000274 | 22.8775 | | 97.0037 | 0.000029 | 30.4 | | 0.029 | 1002 | | 0.60 | 1.15 | | 0.00070 | 22.8770 | | 97.0037 | 0.000029 | 30.4 | | 0.029 | 1002 | | 0.60 | 1.15 | | 0.00070 |
| 51 | 98.5 | 0.0000282 | 24.8775 | | 99.0141 | 0.000030 | 30.4 | | 0.029 | 1002 | | 0.60 | 1.15 | | 0.00070 | 24.8771 | | 99.0141 | 0.000030 | 30.4 | | 0.029 | 1002 | | 0.60 | 1.15 | | 0.00070 |
| 52 | 100.5 | 0.0000268 | 26.8766 | | 101.0246 | 0.000028 | 30.3 | | 0.029 | 1002 | | 0.60 | 1.15 | | 0.00070 | 26.8760 | | 101.0246 | 0.000028 | 30.4 | | 0.029 | 1002 | | 0.60 | 1.15 | | 0.00070 |
| 53 | 102.5 | 0.0000276 | 28.8765 | | 103.0350 | 0.000029 | 30.3 | | 0.029 | 1002 | | 0.60 | 1.15 | | 0.00070 | 28.8761 | | 103.0350 | 0.000029 | 30.3 | | 0.029 | 1002 | | 0.60 | 1.15 | | 0.00070 |
| 54 | 104.5 | 0.0000284 | 30.8766 | | 105.0455 | 0.000030 | 30.3 | | 0.029 | 1002 | | 0.60 | 1.15 | | 0.00070 | 30.8758 | | 105.0455 | 0.000030 | 30.3 | | 0.029 | 1002 | | 0.60 | 1.15 | | 0.00070 |
| 55 | 106.5 | 0.0000286 | 32.8764 | | 107.0559 | 0.000030 | 30.3 | | 0.029 | 1002 | | 0.60 | 1.15 | | 0.00070 | 32.8759 | | 107.0559 | 0.000030 | 30.3 | | 0.029 | 1002 | | 0.60 | 1.15 | | 0.00070 |
| 56 | 108.5 | 0.0000293 | 34.8765 | | 109.0663 | 0.000031 | 30.3 | | 0.029 | 1002 | | 0.60 | 1.15 | | 0.00070 | 34.8760 | | 109.0663 | 0.000031 | 30.3 | | 0.029 | 1002 | | 0.60 | 1.15 | | 0.00070 |
| 57 | 110.5 | 0.0000286 | 36.8765 | | 111.0768 | 0.000030 | 30.3 | | 0.029 | 1002 | | 0.60 | 1.15 | | 0.00070 | 36.8756 | | 111.0768 | 0.000030 | 30.3 | | 0.029 | 1002 | | 0.60 | 1.15 | | 0.00070 |
| 58 | 112.5 | 0.0000294 | 38.8766 | | 113.0872 | 0.000031 | 30.3 | | 0.029 | 1002 | | 0.60 | 1.15 | | 0.00070 | 38.8757 | | 113.0872 | 0.000031 | 30.3 | | 0.029 | 1002 | | 0.60 | 1.15 | | 0.00070 |
| 59 | 114.5 | 0.0000301 | 40.8766 | | 115.0976 | 0.000032 | 30.3 | | 0.029 | 1002 | | 0.60 | 1.15 | | 0.00070 | 40.8757 | | 115.0976 | 0.000032 | 30.2 | | 0.029 | 1002 | | 0.60 | 1.15 | | 0.00070 |
| 60 | 116.5 | 0.0000303 | 42.8766 | | 117.1081 | 0.000032 | 30.3 | | 0.029 | 1002 | | 0.60 | 1.15 | | 0.00070 | 42.8758 | | 117.1081 | 0.000032 | 30.2 | | 0.029 | 1002 | | 0.60 | 1.15 | | 0.00070 |
| 61 | 118.5 | 0.0000310 | 44.8765 | | 119.1185 | 0.000033 | 30.3 | | 0.029 | 1002 | | 0.60 | 1.15 | | 0.00070 | 44.8762 | | 119.1185 | 0.000033 | 30.2 | | 0.029 | 1002 | | 0.60 | 1.15 | | 0.00070 |
| 62 | 120.5 | 0.0000300 | 46.8766 | | 121.1290 | 0.000032 | 30.3 | | 0.029 | 1002 | | 0.60 | 1.15 | | 0.00070 | 46.8758 | | 121.1290 | 0.000032 | 30.2 | | 0.029 | 1002 | | 0.60 | 1.15 | | 0.00070 |
| 63 | 122.5 | 0.0000307 | 48.8765 | | 123.1394 | 0.000033 | 30.3 | | 0.029 | 1002 | | 0.60 | 1.15 | | 0.00070 | 48.8757 | | 123.1394 | 0.000033 | 30.2 | | 0.029 | 1002 | | 0.60 | 1.15 | | 0.00070 |
| 64 | 124.5 | 0.0000314 | 50.8763 | | 125.1498 | 0.000033 | 30.3 | | 0.029 | 1002 | | 0.60 | 1.15 | | 0.00070 | 50.8758 | | 125.1498 | 0.000033 | 30.2 | | 0.029 | 1002 | | 0.60 | 1.15 | | 0.00070 |
| 65 | 126.5 | 0.0000316 | 52.8763 | | 127.1603 | 0.000034 | 30.2 | | 0.029 | 1002 | | 0.60 | 1.15 | | 0.00070 | 52.8759 | | 127.1603 | 0.000034 | 30.2 | | 0.029 | 1002 | | 0.60 | 1.15 | | 0.00070 |
| 66 | 128.5 | 0.0000323 | 54.8763 | | 129.1707 | 0.000034 | 30.2 | | 0.029 | 1002 | | 0.60 | 1.15 | | 0.00070 | 54.8761 | | 129.1707 | 0.000034 | 30.2 | | 0.029 | 1002 | | 0.60 | 1.15 | | 0.00070 |
| 67 | 130.5 | 0.0000317 | 56.8762 | | 131.1812 | 0.000034 | 30.2 | | 0.029 | 1002 | | 0.60 | 1.15 | | 0.00070 | 56.8756 | | 131.1812 | 0.000034 | 30.2 | | 0.029 | 1002 | | 0.60 | 1.15 | | 0.00070 |
| 68 | 132.5 | 0.0000323 | 58.8761 | | 133.1916 | 0.000034 | 30.2 | | 0.029 | 1002 | | 0.60 | 1.15 | | 0.00070 | 58.8754 | | 133.1916 | 0.000034 | 30.2 | | 0.029 | 1002 | | 0.60 | 1.15 | | 0.00070 |
| 69 | 134.5 | 0.0000330 | 60.8762 | | 135.2020 | 0.000035 | 30.2 | | 0.029 | 1002 | | 0.60 | 1.15 | | 0.00070 | 60.8756 | | 135.2020 | 0.000035 | 30.2 | | 0.029 | 1002 | | 0.60 | 1.15 | | 0.00070 |
| 70 | 136.5 | 0.0000332 | 62.8762 | | 137.2125 | 0.000035 | 30.2 | | 0.029 | 1002 | | 0.60 | 1.15 | | 0.00070 | 62.8754 | | 137.2125 | 0.000035 | 30.2 | | 0.029 | 1002 | | 0.60 | 1.15 | | 0.00070 |
| 71 | 138.5 | 0.0000338 | 64.8760 | | 139.2229 | 0.000036 | 30.2 | | 0.029 | 1002 | | 0.60 | 1.15 | | 0.00070 | 64.8754 | | 139.2229 | 0.000036 | 30.2 | | 0.029 | 1002 | | 0.60 | 1.15 | | 0.00070 |
| 72 | 140.5 | 0.0000329 | 66.8758 | | 141.2334 | 0.000035 | 30.2 | | 0.029 | 1002 | | 0.60 | 1.15 | | 0.00070 | 66.8758 | | 141.2334 | 0.000035 | 30.2 | | 0.029 | 1002 | | 0.60 | 1.15 | | 0.00070 |
| 73 | 142.5 | 0.0000336 | 68.8762 | | 143.2438 | 0.000036 | 30.2 | | 0.029 | 1002 | | 0.60 | 1.15 | | 0.00070 | 68.8758 | | 143.2438 | 0.000036 | 30.2 | | 0.029 | 1002 | | 0.60 | 1.15 | | 0.00070 |
| 74 | 144.5 | 0.0000342 | 70.8762 | | 145.2542 | 0.000037 | 30.2 | | 0.029 | 1002 | | 0.60 | 1.15 | | 0.00070 | 70.8756 | | 145.2542 | 0.000037 | 30.2 | | 0.029 | 1002 | | 0.60 | 1.15 | | 0.00070 |
| 75 | 146.5 | 0.0000344 | 72.8760 | | 147.2647 | 0.000037 | 30.2 | | 0.029 | 1002 | | 0.60 | 1.15 | | 0.00070 | 72.8755 | | 147.2647 | 0.000037 | 30.2 | | 0.029 | 1002 | | 0.60 | 1.15 | | 0.00070 |
| 76 | 148.5 | 0.0000350 | 74.8759 | | 149.2751 | 0.000037 | 30.2 | | 0.029 | 1002 | | 0.60 | 1.15 | | 0.00070 | 74.8754 | | 149.2751 | 0.000037 | 30.2 | | 0.029 | 1002 | | 0.60 | 1.15 | | 0.00070 |
| 77 | 150.5 | 0.0000315 | 76.8761 | | 151.2856 | 0.000034 | 30.2 | | 0.029 | 1002 | | 0.60 | 1.15 | | 0.00070 | 76.8755 | | 151.2856 | 0.000034 | 30.2 | | 0.029 | 1002 | | 0.60 | 1.15 | | 0.00070 |
| 78 | 152.5 | 0.0000322 | 78.8759 | | 153.2960 | 0.000035 | 30.2 | | 0.029 | 1002 | | 0.60 | 1.15 | | 0.00070 | 78.8757 | | 153.2960 | 0.000035 | 30.2 | | 0.029 | 1002 | | 0.60 | 1.15 | | 0.00070 |
| 79 | 154.5 | 0.0000328 | 80.8761 | | 155.3064 | 0.000036 | 30.2 | | 0.029 | 1002 | | 0.60 | 1.15 | | 0.00070 | 80.8757 | | 155.3064 | 0.000036 | 30.2 | | 0.029 | 1002 | | 0.60 | 1.15 | | 0.00070 |
| 80 | 156.5 | 0.0000330 | 82.8760 | | 157.3169 | 0.000036 | 30.2 | | 0.029 | 1002 | | 0.60 | 1.15 | | 0.00070 | 82.8757 | | 157.3169 | 0.000036 | 30.2 | | 0.029 | 1002 | | 0.60 | 1.15 | | 0.00070 |
| 81 | 158.5 | 0.0000336 | 84.8760 | | 159.3273 | 0.000037 | 30.2 | | 0.029 | 1002 | | 0.60 | 1.15 | | 0.00070 | 84.8752 | | 159.3273 | 0.000037 | 30.2 | | 0.029 | 1002 | | 0.60 | 1.15 | | 0.00070 |
| 82 | 160.5 | 0.0000330 | 86.8758 | | 161.3377 | 0.000036 | 30.2 | | 0.029 | 1002 | | 0.60 | 1.15 | | 0.00070 | 86.8758 | | 161.3377 | 0.000036 | 30.2 | | 0.029 | 1002 | | 0.60 | 1.15 | | 0.00070 |
| 83 | 162.5 | 0.0000337 | 88.8759 | | 163.3482 | 0.000037 | 30.2 | | 0.029 | 1002 | | 0.60 | 1.15 | | 0.00070 | 88.8755 | | 163.3482 | 0.000037 | 30.2 | | 0.029 | 1002 | | 0.60 | 1.15 | | 0.00070 |
| 84 | 164.5 | 0.0000343 | 90.8758 | | 165.3586 | 0.000037 | 30.2 | | 0.029 | 1002 | | 0.60 | 1.15 | | 0.00070 | 90.8755 | | 165.3586 | 0.000037 | 30.2 | | 0.029 | 1002 | | 0.60 | 1.15 | | 0.00070 |
| 85 | 166.5 | 0.0000345 | 92.8757 | | 167.3691 | 0.000038 | 30.2 | | 0.029 | 1002 | | 0.60 | 1.15 | | 0.00070 | 92.8755 | | 167.3691 | 0.000038 | 30.2 | | 0.029 | 1002 | | 0.60 | 1.15 | | 0.00070 |
| 86 | 168.5 | 0.0000351 | 94.8757 | | 169.3795 | 0.000038 | 30.2 | | 0.029 | 1002 | | 0.60 | 1.15 | | 0.00070 | 94.8753 | | 169.3795 | 0.000038 | 30.2 | | 0.029 | 1002 | | 0.60 | 1.15 | | 0.00070 |
| 87 | 170.5 | 0.0000343 | 96.8760 | | 171.3899 | 0.000038 | 30.2 | | 0.029 | 1002 | | 0.60 | 1.15 | | 0.00070 | 96.8753 | | 171.3899 | 0.000038 | 30.2 | | 0.029 | 1002 | | 0.60 | 1.15 | | 0.00070 |
| 88 | 172.5 | 0.0000349 | 98.8762 | | 173.4004 | 0.000038 | 30.2 | | 0.029 | 1002 | | 0.60 | 1.15 | | 0.00070 | 98.8755 | | 173.4004 | 0.000038 | 30.2 | | 0.029 | 1002 | | 0.60 | 1.15 | | 0.00070 |
| 89 | 174.5 | 0.0000355 | 100.8760 | | 175.4108 | 0.000039 | 30.2 | | 0.029 | 1002 | | 0.60 | 1.15 | | 0.00070 | 100.8754 | | 175.4108 | 0.000039 | 30.2 | | 0.029 | 1002 | | 0.60 | 1.15 | | 0.00070 |
| 90 | 176.5 | 0.0000357 | 102.8760 | | 177.4213 | 0.000039 | 30.2 | | 0.029 | 1002 | | 0.60 | 1.15 | | 0.00070 | 102.8753 | | 177.4213 | 0.000039 | 30.2 | | 0.029 | 1002 | | 0.60 | 1.15 | | 0.00070 |
| 91 | 178.5 | 0.0000362 | 104.8759 | | 179.4317 | 0.000040 | 30.2 | | 0.029 | 1002 | | 0.60 | 1.15 | | 0.00070 | 104.8754 | | 179.4317 | 0.000040 | 30.2 | | 0.029 | 1002 | | 0.60 | 1.15 | | 0.00070 |
| 92 | 180.5 | 0.0000357 | 106.8757 | | 181.4421 | 0.000039 | 30.1 | | 0.029 | 1002 | | 0.60 | 1.15 | | 0.00070 | 106.8753 | | 181.4421 | 0.000039 | 30.2 | | 0.029 | 1002 | | 0.60 | 1.15 | | 0.00070 |
| 93 | 182.5 | 0.0000363 | 108.8760 | | 183.4526 | 0.000040 | 30.1 | | 0.029 | 1002 | | 0.60 | 1.15 | | 0.00070 | 108.8753 | | 183.4526 | 0.000040 | 30.2 | | 0.029 | 1002 | | 0.60 | 1.15 | | 0.00070 |
| 94 | 184.5 | 0.0000369 | 110.8761 | | 185.4630 | 0.000040 | 30.1 | | 0.029 | 1002 | | 0.60 | 1.15 | | 0.00070 | 110.8750 | | 185.4630 | 0.000040 | 30.2 | | 0.029 | 1002 | | 0.60 | 1.15 | | 0.00070 |
| 95 | 186.5 | 0.0000370 | 112.8760 | | 187.4735 | 0.000041 | 30.1 | | 0.029 | 1002 | | 0.60 | 1.15 | | 0.00070 | 112.8752 | | 187.4735 | 0.000041 | 30.2 | | 0.029 | 1002 | | 0.60 | 1.15 | | 0.00070 |
| 96 | 188.5 | 0.0000376 | 114.8761 | | 189.4839 | 0.000041 | 30.1 | | 0.029 | 1002 | | 0.60 | 1.15 | | 0.00070 | 114.8753 | | 189.4839 | 0.000041 | 30.2 | | 0.029 | 1002 | | 0.60 | 1.15 | | 0.00070 |
| 97 | 190.5 | 0.0000368 | 116.8759 | | 191.4943 | 0.000041 | 30.1 | | 0.029 | 1002 | | 0.60 | 1.15 | | 0.00070 | 116.8751 | | 191.4943 | 0.000041 | 30.2 | | 0.029 | 1002 | | 0.60 | 1.15 | | 0.00070 |
| 98 | 192.5 | 0.0000371 | 118.8758 | | 193.5048 | 0.000041 | 30.1 | | 0.029 | 1002 | | 0.60 | 1.15 | | 0.00070 | 118.8755 | | 193.5048 | 0.000041 | 30.2 | | 0.029 | 1002 | | 0.60 | 1.15 | | 0.00070 |
| 99 | 194.5 | 0.0000376 | 120.8759 | | 195.5152 | 0.000041 | 30.1 | | 0.029 | 1002 | | 0.60 | 1.15 | | 0.00070 | 120.8755 | | 195.5152 | 0.000041 | 30.2 | | 0.029 | 1002 | | 0.60 | 1.15 | | 0.00070 |
| 100 | 196.5 | 0.0000381 | 122.8757 | | 197.5257 | 0.000042 | 30.1 | | 0.029 | 1002 | | 0.60 | 1.15 | | 0.00070 | 122.8755 | | 197.5257 | 0.000042 | 30.2 | | 0.029 | 1002 | | 0.60 | 1.15 | | 0.00070 |
| 101 | 198.5 | 0.0000387 | 124.8759 | | 199.5361 | 0.000043 | 30.1 | | 0.029 | 1002 | | 0.60 | 1.15 | | 0.00070 | 124.8758 | | 199.5361 | 0.000043 | 30.2 | | 0.029 | 1002 | | 0.60 | 1.15 | | 0.00070 |
| 102 | 200.5 | 0.0000502 | 126.8753 | | 201.5465 | 0.000053 | 30.1 | | 0.029 | 1002 | | 0.60 | 1.15 | | 0.00070 | 126.8753 | | 201.5465 | 0.000053 | 30.1 | | 0.029 | 1002 | | 0.60 | 1.15 | | 0.00070 |
