# Supplementary material for: Evaluation of the metrological reliability of a graduated cylinder from experimental data from an in-situ calibration
Source: Data Brief. 2020 Aug 6;32:106133. doi: 10.1016/j.dib.2020.106133 (PMC7406472; doi:10.1016/j.dib.2020.106133)
Supplement: Supplementary file 3 [file mmc3.docx]

**Table 5** – Experimental Data (without applying the Tare Function)

| **Exp. Point** | **Standard Mass** | | | **ASCENDING LOAD** | | | | | | | | | | | | | **DESCENDING LOAD** | | | | | | | | | | | |
| --- | --- | --- | --- | --- | --- | --- | --- | --- | --- | --- | --- | --- | --- | --- | --- | --- | --- | --- | --- | --- | --- | --- | --- | --- | --- | --- | --- | --- |
|  |  |  |  | **Analytical Scale** | | | | **Environmental Temperature** | | | **Atmospheric Pressure** | | | **Air Density** | | | **Analytical Scale** | | | | **Environmental Temperature** | | | **Atmospheric Pressure** | | | **Air Density** | |
|  | **Mass** | **Uncertainty** | **Indicated Mass** | | **Apparent Mass** | **Uncertainty of Apparent Mass** | **Indicated Temperature** | | **Uncertainty** | **Indicated Pressure** | | **Uncertainty** | **Air density calculated** | | **Uncertainty** | **Indicated Mass** | | **Apparent Mass** | **Uncertainty of Apparent Mass** | **Indicated Temperature** | | **Uncertainty** | **Indicated Pressure** | | **Uncertainty** | **Air density calculated** | | **Uncertainty** |
|  | **g** | **g** | **g** | | **g** | **g** | **^o^C** | | **^o^C** | **mbar/abs** | | **mbar/abs** | **kg/m^3^** | | **kg/m^3^** | **g** | | **g** | **g** | **^o^C** | | **^o^C** | **mbar/abs** | | **mbar/abs** | **kg/m^3^** | | **kg/m^3^** |
| 1 | 0.0 | 0.0000000 | 0.0022 | | 0.0000 | 0.000000 | 28.0 | | 0.029 | 1002 | | 0.60 | 1.16 | | 0.00070 | 0.0024 | | 0.0000 | 0.000000 | 29.8 | | 0.029 | 1000 | | 0.60 | 1.15 | | 0.00070 |
| 2 | 0.5 | 0.0000042 | 0.5022 | | 0.5026 | 0.000004 | 28.1 | | 0.029 | 1002 | | 0.60 | 1.16 | | 0.00070 | 0.5024 | | 0.5026 | 0.000004 | 29.7 | | 0.029 | 1000 | | 0.60 | 1.15 | | 0.00070 |
| 3 | 2.5 | 0.0000077 | 2.5022 | | 2.5130 | 0.000008 | 28.2 | | 0.029 | 1002 | | 0.60 | 1.16 | | 0.00070 | 2.5026 | | 2.5130 | 0.000008 | 29.7 | | 0.029 | 1000 | | 0.60 | 1.15 | | 0.00070 |
| 4 | 4.5 | 0.0000101 | 4.5022 | | 4.5235 | 0.000010 | 28.2 | | 0.029 | 1002 | | 0.60 | 1.16 | | 0.00070 | 4.5025 | | 4.5235 | 0.000010 | 29.7 | | 0.029 | 1000 | | 0.60 | 1.15 | | 0.00070 |
| 5 | 6.5 | 0.0000107 | 6.5024 | | 6.5339 | 0.000011 | 28.4 | | 0.029 | 1002 | | 0.60 | 1.16 | | 0.00070 | 6.5027 | | 6.5339 | 0.000011 | 29.7 | | 0.029 | 1000 | | 0.60 | 1.15 | | 0.00070 |
| 6 | 8.5 | 0.0000125 | 8.5024 | | 8.5444 | 0.000013 | 28.4 | | 0.029 | 1002 | | 0.60 | 1.16 | | 0.00070 | 8.5026 | | 8.5444 | 0.000013 | 29.7 | | 0.029 | 1000 | | 0.60 | 1.15 | | 0.00070 |
| 7 | 10.5 | 0.0000108 | 10.5023 | | 10.5548 | 0.000011 | 28.4 | | 0.029 | 1002 | | 0.60 | 1.16 | | 0.00070 | 10.5029 | | 10.5548 | 0.000011 | 29.7 | | 0.029 | 1000 | | 0.60 | 1.15 | | 0.00070 |
| 8 | 12.5 | 0.0000126 | 12.5023 | | 12.5652 | 0.000013 | 28.4 | | 0.029 | 1002 | | 0.60 | 1.16 | | 0.00070 | 12.5028 | | 12.5652 | 0.000013 | 29.7 | | 0.029 | 1000 | | 0.60 | 1.15 | | 0.00070 |
| 9 | 14.5 | 0.0000142 | 14.5024 | | 14.5757 | 0.000014 | 28.4 | | 0.029 | 1002 | | 0.60 | 1.16 | | 0.00070 | 14.5029 | | 14.5757 | 0.000014 | 29.7 | | 0.029 | 1000 | | 0.60 | 1.15 | | 0.00070 |
| 10 | 16.5 | 0.0000146 | 16.5023 | | 16.5861 | 0.000015 | 28.4 | | 0.029 | 1002 | | 0.60 | 1.16 | | 0.00070 | 16.5029 | | 16.5861 | 0.000015 | 29.7 | | 0.029 | 1000 | | 0.60 | 1.15 | | 0.00070 |
| 11 | 18.5 | 0.0000160 | 18.5025 | | 18.5966 | 0.000016 | 28.4 | | 0.029 | 1002 | | 0.60 | 1.16 | | 0.00070 | 18.5029 | | 18.5966 | 0.000016 | 29.7 | | 0.029 | 1000 | | 0.60 | 1.15 | | 0.00070 |
| 12 | 20.5 | 0.0000141 | 20.5025 | | 20.6070 | 0.000014 | 28.5 | | 0.029 | 1002 | | 0.60 | 1.16 | | 0.00070 | 20.5028 | | 20.6070 | 0.000014 | 29.7 | | 0.029 | 1000 | | 0.60 | 1.15 | | 0.00070 |
| 13 | 22.5 | 0.0000155 | 22.5027 | | 22.6174 | 0.000016 | 28.5 | | 0.029 | 1002 | | 0.60 | 1.16 | | 0.00070 | 22.5030 | | 22.6174 | 0.000016 | 29.7 | | 0.029 | 1000 | | 0.60 | 1.15 | | 0.00070 |
| 14 | 24.5 | 0.0000169 | 24.5026 | | 24.6279 | 0.000017 | 28.5 | | 0.029 | 1002 | | 0.60 | 1.16 | | 0.00070 | 24.5029 | | 24.6279 | 0.000017 | 29.7 | | 0.029 | 1000 | | 0.60 | 1.15 | | 0.00070 |
| 15 | 26.5 | 0.0000167 | 26.5027 | | 26.6383 | 0.000017 | 28.5 | | 0.029 | 1002 | | 0.60 | 1.16 | | 0.00070 | 26.5029 | | 26.6383 | 0.000017 | 29.7 | | 0.029 | 1000 | | 0.60 | 1.15 | | 0.00070 |
| 16 | 28.5 | 0.0000179 | 28.5028 | | 28.6487 | 0.000018 | 28.5 | | 0.029 | 1002 | | 0.60 | 1.16 | | 0.00070 | 28.5033 | | 28.6487 | 0.000018 | 29.7 | | 0.029 | 1000 | | 0.60 | 1.15 | | 0.00070 |
| 17 | 30.5 | 0.0000173 | 30.5028 | | 30.6592 | 0.000018 | 28.5 | | 0.029 | 1002 | | 0.60 | 1.16 | | 0.00070 | 30.5031 | | 30.6592 | 0.000018 | 29.7 | | 0.029 | 1000 | | 0.60 | 1.15 | | 0.00070 |
| 18 | 32.5 | 0.0000185 | 32.5028 | | 32.6696 | 0.000019 | 28.5 | | 0.029 | 1002 | | 0.60 | 1.16 | | 0.00070 | 32.5032 | | 32.6696 | 0.000019 | 29.7 | | 0.029 | 1000 | | 0.60 | 1.15 | | 0.00070 |
| 19 | 34.5 | 0.0000196 | 34.5028 | | 34.6801 | 0.000020 | 28.6 | | 0.029 | 1002 | | 0.60 | 1.16 | | 0.00070 | 34.5030 | | 34.6801 | 0.000020 | 29.7 | | 0.029 | 1000 | | 0.60 | 1.15 | | 0.00070 |
| 20 | 36.5 | 0.0000199 | 36.5028 | | 36.6905 | 0.000020 | 28.6 | | 0.029 | 1002 | | 0.60 | 1.16 | | 0.00070 | 36.5030 | | 36.6905 | 0.000020 | 29.7 | | 0.029 | 1000 | | 0.60 | 1.15 | | 0.00070 |
| 21 | 38.5 | 0.0000210 | 38.5028 | | 38.7009 | 0.000021 | 28.6 | | 0.029 | 1002 | | 0.60 | 1.16 | | 0.00070 | 38.5034 | | 38.7009 | 0.000021 | 29.7 | | 0.029 | 1000 | | 0.60 | 1.15 | | 0.00070 |
| 22 | 40.5 | 0.0000195 | 40.5027 | | 40.7114 | 0.000020 | 28.7 | | 0.029 | 1002 | | 0.60 | 1.16 | | 0.00070 | 40.5031 | | 40.7114 | 0.000020 | 29.7 | | 0.029 | 1000 | | 0.60 | 1.15 | | 0.00070 |
| 23 | 42.5 | 0.0000206 | 42.5028 | | 42.7218 | 0.000021 | 28.7 | | 0.029 | 1002 | | 0.60 | 1.16 | | 0.00070 | 42.5031 | | 42.7218 | 0.000021 | 29.7 | | 0.029 | 1000 | | 0.60 | 1.15 | | 0.00070 |
| 24 | 44.5 | 0.0000216 | 44.5030 | | 44.7322 | 0.000022 | 28.7 | | 0.029 | 1002 | | 0.60 | 1.16 | | 0.00070 | 44.5032 | | 44.7322 | 0.000022 | 29.7 | | 0.029 | 1000 | | 0.60 | 1.15 | | 0.00070 |
| 25 | 46.5 | 0.0000219 | 46.5028 | | 46.7427 | 0.000022 | 28.7 | | 0.029 | 1002 | | 0.60 | 1.16 | | 0.00070 | 46.5032 | | 46.7427 | 0.000022 | 29.7 | | 0.029 | 1000 | | 0.60 | 1.15 | | 0.00070 |
| 26 | 48.5 | 0.0000228 | 48.5030 | | 48.7531 | 0.000023 | 28.7 | | 0.029 | 1002 | | 0.60 | 1.16 | | 0.00070 | 48.5032 | | 48.7531 | 0.000023 | 29.7 | | 0.029 | 1000 | | 0.60 | 1.15 | | 0.00070 |
| 27 | 50.5 | 0.0000170 | 50.5031 | | 50.7636 | 0.000018 | 28.8 | | 0.029 | 1002 | | 0.60 | 1.16 | | 0.00070 | 50.5031 | | 50.7636 | 0.000018 | 29.7 | | 0.029 | 1000 | | 0.60 | 1.15 | | 0.00070 |
| 28 | 52.5 | 0.0000182 | 52.5033 | | 52.7740 | 0.000019 | 28.8 | | 0.029 | 1001 | | 0.60 | 1.16 | | 0.00070 | 52.5031 | | 52.7740 | 0.000019 | 29.7 | | 0.029 | 1000 | | 0.60 | 1.15 | | 0.00070 |
| 29 | 54.5 | 0.0000193 | 54.5033 | | 54.7844 | 0.000020 | 28.8 | | 0.029 | 1001 | | 0.60 | 1.16 | | 0.00070 | 54.5031 | | 54.7844 | 0.000020 | 29.7 | | 0.029 | 1000 | | 0.60 | 1.15 | | 0.00070 |
| 30 | 56.5 | 0.0000197 | 56.5032 | | 56.7949 | 0.000020 | 28.8 | | 0.029 | 1001 | | 0.60 | 1.16 | | 0.00070 | 56.5031 | | 56.7949 | 0.000020 | 29.6 | | 0.029 | 1000 | | 0.60 | 1.15 | | 0.00070 |
| 31 | 58.5 | 0.0000207 | 58.5034 | | 58.8053 | 0.000021 | 28.8 | | 0.029 | 1001 | | 0.60 | 1.16 | | 0.00070 | 58.5029 | | 58.8053 | 0.000021 | 29.6 | | 0.029 | 1000 | | 0.60 | 1.15 | | 0.00070 |
| 32 | 60.5 | 0.0000197 | 60.5030 | | 60.8157 | 0.000021 | 28.8 | | 0.029 | 1001 | | 0.60 | 1.16 | | 0.00070 | 60.5032 | | 60.8157 | 0.000021 | 29.6 | | 0.029 | 1000 | | 0.60 | 1.15 | | 0.00070 |
| 33 | 62.5 | 0.0000208 | 62.5032 | | 62.8262 | 0.000022 | 28.8 | | 0.029 | 1001 | | 0.60 | 1.16 | | 0.00070 | 62.5031 | | 62.8262 | 0.000022 | 29.6 | | 0.029 | 1000 | | 0.60 | 1.15 | | 0.00070 |
| 34 | 64.5 | 0.0000218 | 64.5032 | | 64.8366 | 0.000023 | 28.8 | | 0.029 | 1001 | | 0.60 | 1.16 | | 0.00070 | 64.5033 | | 64.8366 | 0.000023 | 29.6 | | 0.029 | 1000 | | 0.60 | 1.15 | | 0.00070 |
| 35 | 66.5 | 0.0000221 | 66.5031 | | 66.8471 | 0.000023 | 28.8 | | 0.029 | 1001 | | 0.60 | 1.16 | | 0.00070 | 66.5033 | | 66.8471 | 0.000023 | 29.6 | | 0.029 | 1000 | | 0.60 | 1.15 | | 0.00070 |
| 36 | 68.5 | 0.0000230 | 68.5033 | | 68.8575 | 0.000024 | 28.8 | | 0.029 | 1001 | | 0.60 | 1.16 | | 0.00070 | 68.5032 | | 68.8575 | 0.000024 | 29.6 | | 0.029 | 1000 | | 0.60 | 1.15 | | 0.00070 |
| 37 | 70.5 | 0.0000217 | 70.5029 | | 70.8679 | 0.000023 | 28.9 | | 0.029 | 1001 | | 0.60 | 1.15 | | 0.00070 | 70.5035 | | 70.8679 | 0.000023 | 29.6 | | 0.029 | 1000 | | 0.60 | 1.15 | | 0.00070 |
| 38 | 72.5 | 0.0000227 | 72.5031 | | 72.8784 | 0.000024 | 28.9 | | 0.029 | 1001 | | 0.60 | 1.15 | | 0.00070 | 72.5035 | | 72.8784 | 0.000024 | 29.6 | | 0.029 | 1000 | | 0.60 | 1.15 | | 0.00070 |
| 39 | 74.5 | 0.0000236 | 74.5030 | | 74.8888 | 0.000025 | 28.9 | | 0.029 | 1001 | | 0.60 | 1.15 | | 0.00070 | 74.5035 | | 74.8888 | 0.000025 | 29.6 | | 0.029 | 1000 | | 0.60 | 1.15 | | 0.00070 |
| 40 | 76.5 | 0.0000239 | 76.5031 | | 76.8993 | 0.000025 | 28.9 | | 0.029 | 1001 | | 0.60 | 1.15 | | 0.00070 | 76.5036 | | 76.8993 | 0.000025 | 29.6 | | 0.029 | 1000 | | 0.60 | 1.15 | | 0.00070 |
| 41 | 78.5 | 0.0000247 | 78.5032 | | 78.9097 | 0.000026 | 28.9 | | 0.029 | 1001 | | 0.60 | 1.15 | | 0.00070 | 78.5035 | | 78.9097 | 0.000026 | 29.6 | | 0.029 | 1000 | | 0.60 | 1.15 | | 0.00070 |
| 42 | 80.5 | 0.0000239 | 80.5027 | | 80.9201 | 0.000025 | 28.9 | | 0.029 | 1001 | | 0.60 | 1.15 | | 0.00070 | 80.5035 | | 80.9201 | 0.000025 | 29.6 | | 0.029 | 1000 | | 0.60 | 1.15 | | 0.00070 |
| 43 | 82.5 | 0.0000248 | 82.5032 | | 82.9306 | 0.000026 | 28.9 | | 0.029 | 1001 | | 0.60 | 1.15 | | 0.00070 | 82.5035 | | 82.9306 | 0.000026 | 29.6 | | 0.029 | 1000 | | 0.60 | 1.15 | | 0.00070 |
| 44 | 84.5 | 0.0000256 | 84.5031 | | 84.9410 | 0.000027 | 28.9 | | 0.029 | 1001 | | 0.60 | 1.15 | | 0.00070 | 84.5034 | | 84.9410 | 0.000027 | 29.6 | | 0.029 | 1000 | | 0.60 | 1.15 | | 0.00070 |
| 45 | 86.5 | 0.0000239 | 86.5033 | | 86.9514 | 0.000025 | 28.9 | | 0.029 | 1001 | | 0.60 | 1.15 | | 0.00070 | 86.5036 | | 86.9514 | 0.000025 | 29.6 | | 0.029 | 1000 | | 0.60 | 1.15 | | 0.00070 |
| 46 | 88.5 | 0.0000247 | 88.5031 | | 88.9619 | 0.000026 | 28.9 | | 0.029 | 1001 | | 0.60 | 1.15 | | 0.00070 | 88.5036 | | 88.9619 | 0.000026 | 29.6 | | 0.029 | 1000 | | 0.60 | 1.15 | | 0.00070 |
| 47 | 90.5 | 0.0000256 | 90.5033 | | 90.9723 | 0.000027 | 29.0 | | 0.029 | 1001 | | 0.60 | 1.15 | | 0.00070 | 90.5035 | | 90.9723 | 0.000027 | 29.6 | | 0.029 | 1000 | | 0.60 | 1.15 | | 0.00070 |
| 48 | 92.5 | 0.0000264 | 92.5033 | | 92.9828 | 0.000028 | 29.0 | | 0.029 | 1001 | | 0.60 | 1.15 | | 0.00070 | 92.5035 | | 92.9828 | 0.000028 | 29.6 | | 0.029 | 1000 | | 0.60 | 1.15 | | 0.00070 |
| 49 | 94.5 | 0.0000272 | 94.5033 | | 94.9932 | 0.000029 | 29.0 | | 0.029 | 1001 | | 0.60 | 1.15 | | 0.00070 | 94.5035 | | 94.9932 | 0.000029 | 29.6 | | 0.029 | 1000 | | 0.60 | 1.15 | | 0.00070 |
| 50 | 96.5 | 0.0000274 | 96.5037 | | 97.0036 | 0.000029 | 29.1 | | 0.029 | 1001 | | 0.60 | 1.15 | | 0.00070 | 96.5037 | | 97.0036 | 0.000029 | 29.6 | | 0.029 | 1000 | | 0.60 | 1.15 | | 0.00070 |
| 51 | 98.5 | 0.0000282 | 98.5038 | | 99.0141 | 0.000030 | 29.1 | | 0.029 | 1001 | | 0.60 | 1.15 | | 0.00070 | 98.5037 | | 99.0141 | 0.000030 | 29.6 | | 0.029 | 1000 | | 0.60 | 1.15 | | 0.00070 |
| 52 | 100.5 | 0.0000268 | 100.5025 | | 101.0245 | 0.000028 | 29.1 | | 0.029 | 1001 | | 0.60 | 1.15 | | 0.00070 | 100.5031 | | 101.0245 | 0.000028 | 29.6 | | 0.029 | 1000 | | 0.60 | 1.15 | | 0.00070 |
| 53 | 102.5 | 0.0000276 | 102.5027 | | 103.0349 | 0.000029 | 29.1 | | 0.029 | 1001 | | 0.60 | 1.15 | | 0.00070 | 102.5030 | | 103.0349 | 0.000029 | 29.6 | | 0.029 | 1000 | | 0.60 | 1.15 | | 0.00070 |
| 54 | 104.5 | 0.0000284 | 104.5027 | | 105.0454 | 0.000030 | 29.1 | | 0.029 | 1001 | | 0.60 | 1.15 | | 0.00070 | 104.5029 | | 105.0454 | 0.000030 | 29.6 | | 0.029 | 1000 | | 0.60 | 1.15 | | 0.00070 |
| 55 | 106.5 | 0.0000286 | 106.5025 | | 107.0558 | 0.000030 | 29.1 | | 0.029 | 1001 | | 0.60 | 1.15 | | 0.00070 | 106.5028 | | 107.0558 | 0.000030 | 29.6 | | 0.029 | 1000 | | 0.60 | 1.15 | | 0.00070 |
| 56 | 108.5 | 0.0000293 | 108.5026 | | 109.0663 | 0.000031 | 29.1 | | 0.029 | 1001 | | 0.60 | 1.15 | | 0.00070 | 108.5028 | | 109.0663 | 0.000031 | 29.6 | | 0.029 | 1000 | | 0.60 | 1.15 | | 0.00070 |
| 57 | 110.5 | 0.0000286 | 110.5026 | | 111.0767 | 0.000030 | 29.1 | | 0.029 | 1001 | | 0.60 | 1.15 | | 0.00070 | 110.5027 | | 111.0767 | 0.000030 | 29.5 | | 0.029 | 1000 | | 0.60 | 1.15 | | 0.00070 |
| 58 | 112.5 | 0.0000294 | 112.5026 | | 113.0871 | 0.000031 | 29.1 | | 0.029 | 1001 | | 0.60 | 1.15 | | 0.00070 | 112.5027 | | 113.0871 | 0.000031 | 29.5 | | 0.029 | 1000 | | 0.60 | 1.15 | | 0.00070 |
| 59 | 114.5 | 0.0000301 | 114.5027 | | 115.0976 | 0.000032 | 29.1 | | 0.029 | 1001 | | 0.60 | 1.15 | | 0.00070 | 114.5028 | | 115.0976 | 0.000032 | 29.5 | | 0.029 | 1000 | | 0.60 | 1.15 | | 0.00070 |
| 60 | 116.5 | 0.0000303 | 116.5026 | | 117.1080 | 0.000032 | 29.1 | | 0.029 | 1001 | | 0.60 | 1.15 | | 0.00070 | 116.5028 | | 117.1080 | 0.000032 | 29.5 | | 0.029 | 1000 | | 0.60 | 1.15 | | 0.00070 |
| 61 | 118.5 | 0.0000310 | 118.5027 | | 119.1184 | 0.000033 | 29.1 | | 0.029 | 1001 | | 0.60 | 1.15 | | 0.00070 | 118.5028 | | 119.1184 | 0.000033 | 29.5 | | 0.029 | 1000 | | 0.60 | 1.15 | | 0.00070 |
| 62 | 120.5 | 0.0000300 | 120.5025 | | 121.1289 | 0.000032 | 29.1 | | 0.029 | 1001 | | 0.60 | 1.15 | | 0.00070 | 120.5027 | | 121.1289 | 0.000032 | 29.5 | | 0.029 | 1000 | | 0.60 | 1.15 | | 0.00070 |
| 63 | 122.5 | 0.0000307 | 122.5027 | | 123.1393 | 0.000033 | 29.1 | | 0.029 | 1001 | | 0.60 | 1.15 | | 0.00070 | 122.5028 | | 123.1393 | 0.000033 | 29.5 | | 0.029 | 1000 | | 0.60 | 1.15 | | 0.00070 |
| 64 | 124.5 | 0.0000314 | 124.5026 | | 125.1498 | 0.000033 | 29.1 | | 0.029 | 1001 | | 0.60 | 1.15 | | 0.00070 | 124.5027 | | 125.1498 | 0.000033 | 29.5 | | 0.029 | 1000 | | 0.60 | 1.15 | | 0.00070 |
| 65 | 126.5 | 0.0000316 | 126.5025 | | 127.1602 | 0.000034 | 29.1 | | 0.029 | 1001 | | 0.60 | 1.15 | | 0.00070 | 126.5027 | | 127.1602 | 0.000034 | 29.5 | | 0.029 | 1000 | | 0.60 | 1.15 | | 0.00070 |
| 66 | 128.5 | 0.0000323 | 128.5026 | | 129.1706 | 0.000034 | 29.1 | | 0.029 | 1001 | | 0.60 | 1.15 | | 0.00070 | 128.5029 | | 129.1706 | 0.000034 | 29.5 | | 0.029 | 1000 | | 0.60 | 1.15 | | 0.00070 |
| 67 | 130.5 | 0.0000317 | 130.5025 | | 131.1811 | 0.000034 | 29.1 | | 0.029 | 1001 | | 0.60 | 1.15 | | 0.00070 | 130.5027 | | 131.1811 | 0.000034 | 29.5 | | 0.029 | 1000 | | 0.60 | 1.15 | | 0.00070 |
| 68 | 132.5 | 0.0000323 | 132.5025 | | 133.1915 | 0.000035 | 29.1 | | 0.029 | 1001 | | 0.60 | 1.15 | | 0.00070 | 132.5027 | | 133.1915 | 0.000035 | 29.5 | | 0.029 | 1000 | | 0.60 | 1.15 | | 0.00070 |
| 69 | 134.5 | 0.0000330 | 134.5026 | | 135.2020 | 0.000035 | 29.1 | | 0.029 | 1001 | | 0.60 | 1.15 | | 0.00070 | 134.5026 | | 135.2020 | 0.000035 | 29.5 | | 0.029 | 1000 | | 0.60 | 1.15 | | 0.00070 |
| 70 | 136.5 | 0.0000332 | 136.5026 | | 137.2124 | 0.000035 | 29.1 | | 0.029 | 1001 | | 0.60 | 1.15 | | 0.00070 | 136.5028 | | 137.2124 | 0.000035 | 29.5 | | 0.029 | 1000 | | 0.60 | 1.15 | | 0.00070 |
| 71 | 138.5 | 0.0000338 | 138.5027 | | 139.2228 | 0.000036 | 29.1 | | 0.029 | 1001 | | 0.60 | 1.15 | | 0.00070 | 138.5028 | | 139.2228 | 0.000036 | 29.5 | | 0.029 | 1000 | | 0.60 | 1.15 | | 0.00070 |
| 72 | 140.5 | 0.0000329 | 140.5026 | | 141.2333 | 0.000035 | 29.1 | | 0.029 | 1001 | | 0.60 | 1.15 | | 0.00070 | 140.5028 | | 141.2333 | 0.000035 | 29.5 | | 0.029 | 1000 | | 0.60 | 1.15 | | 0.00070 |
| 73 | 142.5 | 0.0000336 | 142.5028 | | 143.2437 | 0.000036 | 29.2 | | 0.029 | 1001 | | 0.60 | 1.15 | | 0.00070 | 142.5028 | | 143.2437 | 0.000036 | 29.5 | | 0.029 | 1000 | | 0.60 | 1.15 | | 0.00070 |
| 74 | 144.5 | 0.0000342 | 144.5027 | | 145.2541 | 0.000037 | 29.2 | | 0.029 | 1001 | | 0.60 | 1.15 | | 0.00070 | 144.5028 | | 145.2541 | 0.000037 | 29.5 | | 0.029 | 1000 | | 0.60 | 1.15 | | 0.00070 |
| 75 | 146.5 | 0.0000344 | 146.5026 | | 147.2646 | 0.000037 | 29.2 | | 0.029 | 1001 | | 0.60 | 1.15 | | 0.00070 | 146.5028 | | 147.2646 | 0.000037 | 29.5 | | 0.029 | 1000 | | 0.60 | 1.15 | | 0.00070 |
| 76 | 148.5 | 0.0000350 | 148.5028 | | 149.2750 | 0.000038 | 29.2 | | 0.029 | 1001 | | 0.60 | 1.15 | | 0.00070 | 148.5033 | | 149.2750 | 0.000038 | 29.5 | | 0.029 | 1000 | | 0.60 | 1.15 | | 0.00070 |
| 77 | 150.5 | 0.0000315 | 150.5026 | | 151.2855 | 0.000034 | 29.2 | | 0.029 | 1001 | | 0.60 | 1.15 | | 0.00070 | 150.5028 | | 151.2855 | 0.000034 | 29.5 | | 0.029 | 1000 | | 0.60 | 1.15 | | 0.00070 |
| 78 | 152.5 | 0.0000322 | 152.5028 | | 153.2959 | 0.000035 | 29.2 | | 0.029 | 1001 | | 0.60 | 1.15 | | 0.00070 | 152.5027 | | 153.2959 | 0.000035 | 29.5 | | 0.029 | 1000 | | 0.60 | 1.15 | | 0.00070 |
| 79 | 154.5 | 0.0000328 | 154.5028 | | 155.3063 | 0.000036 | 29.2 | | 0.029 | 1000 | | 0.60 | 1.15 | | 0.00070 | 154.5029 | | 155.3063 | 0.000036 | 29.5 | | 0.029 | 1000 | | 0.60 | 1.15 | | 0.00070 |
| 80 | 156.5 | 0.0000330 | 156.5027 | | 157.3168 | 0.000036 | 29.2 | | 0.029 | 1000 | | 0.60 | 1.15 | | 0.00070 | 156.5028 | | 157.3168 | 0.000036 | 29.5 | | 0.029 | 1000 | | 0.60 | 1.15 | | 0.00070 |
| 81 | 158.5 | 0.0000336 | 158.5028 | | 159.3272 | 0.000037 | 29.2 | | 0.029 | 1000 | | 0.60 | 1.15 | | 0.00070 | 158.5028 | | 159.3272 | 0.000037 | 29.5 | | 0.029 | 1000 | | 0.60 | 1.15 | | 0.00070 |
| 82 | 160.5 | 0.0000330 | 160.5028 | | 161.3376 | 0.000036 | 29.2 | | 0.029 | 1000 | | 0.60 | 1.15 | | 0.00070 | 160.5029 | | 161.3376 | 0.000036 | 29.4 | | 0.029 | 1000 | | 0.60 | 1.15 | | 0.00070 |
| 83 | 162.5 | 0.0000337 | 162.5028 | | 163.3481 | 0.000037 | 29.2 | | 0.029 | 1000 | | 0.60 | 1.15 | | 0.00070 | 162.5030 | | 163.3481 | 0.000037 | 29.4 | | 0.029 | 1000 | | 0.60 | 1.15 | | 0.00070 |
| 84 | 164.5 | 0.0000343 | 164.5029 | | 165.3585 | 0.000037 | 29.2 | | 0.029 | 1000 | | 0.60 | 1.15 | | 0.00070 | 164.5029 | | 165.3585 | 0.000037 | 29.4 | | 0.029 | 1000 | | 0.60 | 1.15 | | 0.00070 |
| 85 | 166.5 | 0.0000345 | 166.5028 | | 167.3690 | 0.000038 | 29.2 | | 0.029 | 1000 | | 0.60 | 1.15 | | 0.00070 | 166.5027 | | 167.3690 | 0.000038 | 29.4 | | 0.029 | 1000 | | 0.60 | 1.15 | | 0.00070 |
| 86 | 168.5 | 0.0000351 | 168.5028 | | 169.3794 | 0.000038 | 29.2 | | 0.029 | 1000 | | 0.60 | 1.15 | | 0.00070 | 168.5029 | | 169.3794 | 0.000038 | 29.4 | | 0.029 | 1000 | | 0.60 | 1.15 | | 0.00070 |
| 87 | 170.5 | 0.0000343 | 170.5028 | | 171.3898 | 0.000038 | 29.2 | | 0.029 | 1000 | | 0.60 | 1.15 | | 0.00070 | 170.5027 | | 171.3898 | 0.000038 | 29.4 | | 0.029 | 1000 | | 0.60 | 1.15 | | 0.00070 |
| 88 | 172.5 | 0.0000349 | 172.5028 | | 173.4003 | 0.000038 | 29.2 | | 0.029 | 1000 | | 0.60 | 1.15 | | 0.00070 | 172.5030 | | 173.4003 | 0.000038 | 29.4 | | 0.029 | 1000 | | 0.60 | 1.15 | | 0.00070 |
| 89 | 174.5 | 0.0000355 | 174.5028 | | 175.4107 | 0.000039 | 29.2 | | 0.029 | 1000 | | 0.60 | 1.15 | | 0.00070 | 174.5029 | | 175.4107 | 0.000039 | 29.4 | | 0.029 | 1000 | | 0.60 | 1.15 | | 0.00070 |
| 90 | 176.5 | 0.0000357 | 176.5028 | | 177.4211 | 0.000039 | 29.2 | | 0.029 | 1000 | | 0.60 | 1.15 | | 0.00070 | 176.5029 | | 177.4211 | 0.000039 | 29.4 | | 0.029 | 1000 | | 0.60 | 1.15 | | 0.00070 |
| 91 | 178.5 | 0.0000362 | 178.5031 | | 179.4316 | 0.000040 | 29.2 | | 0.029 | 1000 | | 0.60 | 1.15 | | 0.00070 | 178.5030 | | 179.4316 | 0.000040 | 29.4 | | 0.029 | 1000 | | 0.60 | 1.15 | | 0.00070 |
| 92 | 180.5 | 0.0000357 | 180.5028 | | 181.4420 | 0.000039 | 29.2 | | 0.029 | 1000 | | 0.60 | 1.15 | | 0.00070 | 180.5030 | | 181.4420 | 0.000039 | 29.4 | | 0.029 | 1000 | | 0.60 | 1.15 | | 0.00070 |
| 93 | 182.5 | 0.0000363 | 182.5029 | | 183.4525 | 0.000040 | 29.2 | | 0.029 | 1000 | | 0.60 | 1.15 | | 0.00070 | 182.5028 | | 183.4525 | 0.000040 | 29.4 | | 0.029 | 1000 | | 0.60 | 1.15 | | 0.00070 |
| 94 | 184.5 | 0.0000369 | 184.5028 | | 185.4629 | 0.000040 | 29.2 | | 0.029 | 1000 | | 0.60 | 1.15 | | 0.00070 | 184.5033 | | 185.4629 | 0.000040 | 29.4 | | 0.029 | 1000 | | 0.60 | 1.15 | | 0.00070 |
| 95 | 186.5 | 0.0000370 | 186.5028 | | 187.4733 | 0.000041 | 29.2 | | 0.029 | 1000 | | 0.60 | 1.15 | | 0.00070 | 186.5031 | | 187.4733 | 0.000041 | 29.4 | | 0.029 | 1000 | | 0.60 | 1.15 | | 0.00070 |
| 96 | 188.5 | 0.0000376 | 188.5030 | | 189.4838 | 0.000041 | 29.2 | | 0.029 | 1000 | | 0.60 | 1.15 | | 0.00070 | 188.5032 | | 189.4838 | 0.000041 | 29.4 | | 0.029 | 1000 | | 0.60 | 1.15 | | 0.00070 |
| 97 | 190.5 | 0.0000368 | 190.5030 | | 191.4942 | 0.000041 | 29.2 | | 0.029 | 1000 | | 0.60 | 1.15 | | 0.00070 | 190.5033 | | 191.4942 | 0.000041 | 29.4 | | 0.029 | 1000 | | 0.60 | 1.15 | | 0.00070 |
| 98 | 192.5 | 0.0000371 | 192.5030 | | 193.5047 | 0.000041 | 29.2 | | 0.029 | 1000 | | 0.60 | 1.15 | | 0.00070 | 192.5032 | | 193.5047 | 0.000041 | 29.4 | | 0.029 | 1000 | | 0.60 | 1.15 | | 0.00070 |
| 99 | 194.5 | 0.0000376 | 194.5032 | | 195.5151 | 0.000042 | 29.2 | | 0.029 | 1000 | | 0.60 | 1.15 | | 0.00070 | 194.5033 | | 195.5151 | 0.000042 | 29.4 | | 0.029 | 1000 | | 0.60 | 1.15 | | 0.00070 |
| 100 | 196.5 | 0.0000381 | 196.5031 | | 197.5255 | 0.000042 | 29.2 | | 0.029 | 1000 | | 0.60 | 1.15 | | 0.00070 | 196.5033 | | 197.5255 | 0.000042 | 29.4 | | 0.029 | 1000 | | 0.60 | 1.15 | | 0.00070 |
| 101 | 198.5 | 0.0000387 | 198.5032 | | 199.5360 | 0.000043 | 29.2 | | 0.029 | 1000 | | 0.60 | 1.15 | | 0.00070 | 198.5038 | | 199.5360 | 0.000043 | 29.3 | | 0.029 | 1000 | | 0.60 | 1.15 | | 0.00070 |
| 102 | 200.5 | 0.0000502 | 200.5035 | | 201.5464 | 0.000053 | 29.2 | | 0.029 | 1000 | | 0.60 | 1.15 | | 0.00070 | 200.5035 | | 201.5464 | 0.000053 | 29.2 | | 0.029 | 1000 | | 0.60 | 1.15 | | 0.00070 |
